# Uracil overload triggers global genome instability and dUTPdysgenesis

**DOI:** 10.1093/nar/gkag1072

**Published:** 2026-10-27

**Authors:** Qiao-Ling Yin, Min He, Ke Zhang, Kejing Li, Yeke Wang, Wan-Jing Ding, Yibin Zhu, Cunqi Ye, Dao-Qiong Zheng

**Affiliations:** State Key Laboratory (SKL) of Biobased Transportation Fuel Technology, Ocean College, Zhejiang University, Zhoushan 321006, China; State Key Laboratory (SKL) of Biobased Transportation Fuel Technology, Ocean College, Zhejiang University, Zhoushan 321006, China; College of Life Sciences, Zhejiang University, Hangzhou 310058, China; State Key Laboratory (SKL) of Biobased Transportation Fuel Technology, Ocean College, Zhejiang University, Zhoushan 321006, China; College of Life Sciences, Zhejiang University, Hangzhou 310058, China; State Key Laboratory (SKL) of Biobased Transportation Fuel Technology, Ocean College, Zhejiang University, Zhoushan 321006, China; Institute of Medical Genetics and Development, Key Laboratory of Reproductive Genetics (Ministry of Education) and Department of Reproductive Endocrinology, Women’s Hospital, School of Medicine, Zhejiang University, Hangzhou 310058, China; Zhejiang Provincial Key Laboratory for Cancer Molecular Cell Biology, Life Sciences Institute, Zhejiang University, Hangzhou 310058, China; State Key Laboratory (SKL) of Biobased Transportation Fuel Technology, Ocean College, Zhejiang University, Zhoushan 321006, China

## Abstract

Uracil accumulation in DNA is a pervasive threat to genomic integrity, yet the global dynamics of how uracil overload drives genome instability remain elusive. Using *Saccharomyces cerevisiae* strains with attenuated dUTPase (*DUT1*) expression, we mapped the landscapes of genomic alterations including point mutations, recombination, and aneuploidy. Deletion of the *UNG1* suppressed most induced mutations and loss-of-heterozygosity (LOH), identifying base excision repair as the primary driver of genomic alterations, whereas aneuploidy directly depends on the persistence of intact genomic uracil. Mechanistically, beyond localized bypass of static AP sites by translesion synthesis polymerases, the massive influx of uracil lesions induces a repair bottleneck by depleting Ung1 and Apn1. This resource titration, coupled with downstream processing intermediates, primarily drives mutagenesis at heterologous nonthymine positions. Furthermore, persistent static AP sites preferentially stimulate homologous recombination sub-pathway that yield interstitial LOH, whereas Apn1-mediated processing channels recombination toward terminal LOH. Crucially, we define a novel post-zygotic isolation mechanism, dUTPdysgenesis, where parental wild-type Ung1 unleashes an immediate enzymatic shock against a uracil-preloaded genome upon mating, triggering G1-phase chromosomal shattering and mutations. Collectively, this study transitions the paradigm of uracil mutagenesis from localized errors to a global engine of genome instability, bridging nucleotide metabolism, DNA repair kinetics, and speciation barriers.

## Graphical Abstract

**Graphical Abstract:**
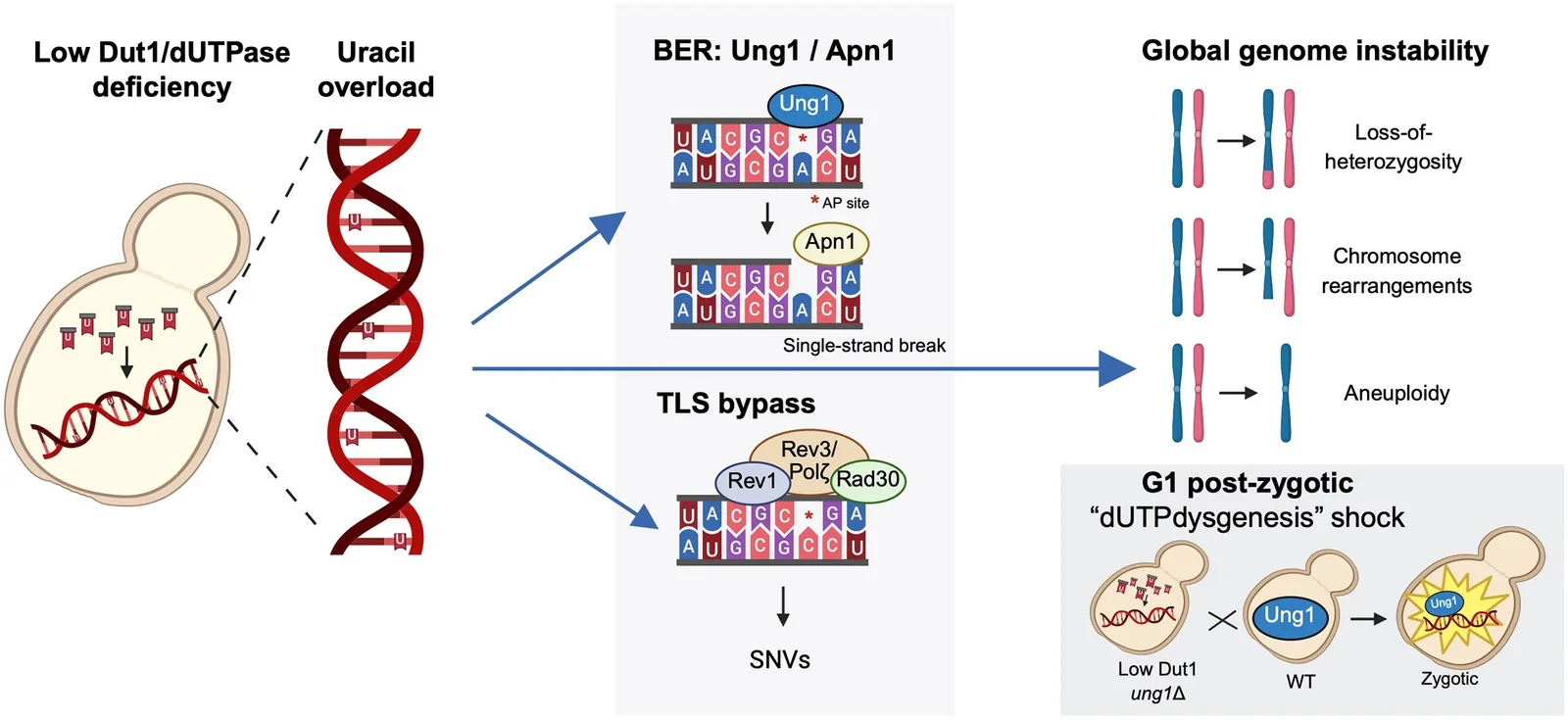

## Introduction

The maintenance of genomic integrity is fundamental to cellular survival and the faithful transmission of genetic information. However, the genome is constantly challenged by endogenous lesions, among which the incorporation of noncanonical or damaged precursor in DNA is one of the most frequent occurrences [1]. To neutralize this risk, organisms rely on a specialized family of nucleotide pool sanitization enzymes [2]. These enzymes, including dUTPase (deoxyuridine 5′-triphosphate nucleotidohydrolase/pyrophosphatase), MTH1 (MutT homolog 1, targeting 8-oxo-dGTP), and ITPA (inosine triphosphate pyrophosphatase, targeting dITP/ITP), selectively hydrolyze aberrant nucleoside triphosphates into their corresponding monophosphates, preventing their mutagenic misincorporation by DNA polymerases. Among these, dUTPase (Dut1 in *Saccharomyces cerevisiae*; EC 3.6.1.23) plays an indispensable role by hydrolyzing dUTP to dUMP (Fig. 1A). Under normal physiological conditions, the misincorporation of dUTP opposite adenine by replicative DNA polymerases during S-phase represents a primary source of genomic uracil, occurring at an estimated rate of 10^4^ events per cell division [1]. Genomic uracil is also generated by spontaneous and enzymatic cytosine deamination, the rate is estimated to be 100–500 cytosines per cell per day [1]. Thanks to robust dUTPase activity and DNA repair surveillance, steady-state genomic uracil is maintained at minimal levels (<1–10 uracil residues per 10^6^ base pairs) [3, 4]. However, under pathological conditions—such as genetic dUTPase deficiency, severe folate deprivation, or treatment with antimetabolite chemotherapeutics like 5-fluorouracil (5-FU)—intracellular dUTP/dTTP ratios surge over 100-fold [5]. While uracil itself is not inherently miscoding, its removal is primarily governed by the base excision repair (BER) [6]. BER is initiated by uracil–DNA glycosylases, such as Ung1 in *S. cerevisiae* or UNG in humans, which catalyze the cleavage of the *N*-glycosidic bond to excise the uracil base, leaving an abasic (AP) site intermediate. Subsequently, an AP endonuclease (such as yeast Apn1 or human APE1) incises the phosphodiester backbone immediately 5′ to the AP site, converting it into a single-strand break (SSB) bearing 3′-hydroxyl and 5′-deoxyribose phosphate termini [7]. Under conditions of uracil overload, high-density uracil excision by Ung1 and subsequent cleavage by Apn1 generate a massive surge of AP sites and SSBs [7]. These intermediates act as formidable barriers to replication forks, leading to fork collapse and the formation of recombinogenic double-strand breaks (DSBs) [7]. Beyond this BER-dependent toxicity, recent studies have unveiled a parallel, excision-independent mechanism: unprocessed genomic uracil, even when escaping the BER pathway, can directly induce replication stress by slowing fork progression [8]. This impediment promotes PrimPol-mediated repriming, leading to the formation of single-stranded DNA (ssDNA) gaps in nascent strands [8].

**Figure 1.**
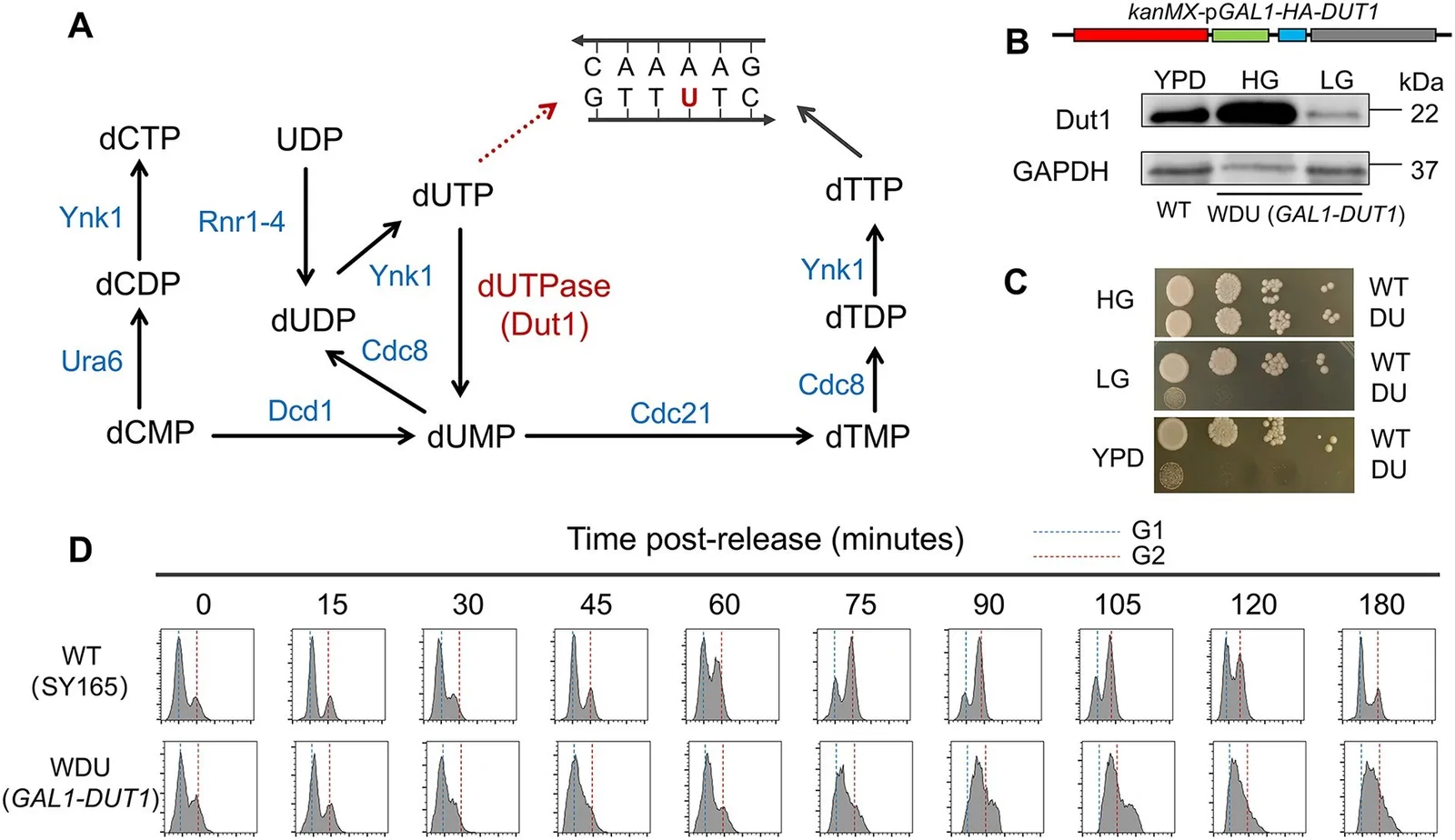
Establishment and validation of a regulatable dUTPase-deficient yeast system. (**A**) Schematic representation of dUTP metabolism in *S. cerevisiae*. dUTPase/Dut1 hydrolyzes dUTP to dUMP, thereby limiting dUTP accumulation and uracil incorporation into DNA. (**B**) Western blot analysis of Dut1 protein levels in wild-type (WT) cells cells grown in extract–peptone–dextrose (YPD) and WDU (*GAL1*-*HA*-*DUT1*) cells cultured under high-galactose (HG; 0.1%) or low-galactose (LG; 0.005%) conditions. GAPDH was used as a loading control. (**C**) Growth of WT and DU (*GAL1-DUT1*) strains on HG, LG, and YPD plates. Ten-fold serial dilutions were spotted onto the indicated solid media and incubated at 30°C. (**D**) Flow cytometry profiles of α-factor-synchronized wild-type (SY165) and WDU cells released into LG medium. Samples were collected at the indicated time points after release. Blue and red dashed lines indicate the approximate G1- and G2-phase DNA content peaks, respectively.

Early pioneering studies in *Escherichia coli* established the critical role of dUTPase in preventing uracil overload. Warner and Duncan first demonstrated that dUTPase deficiency leads to massive uracil incorporation [9], while Tye *et al*. revealed that the resulting DNA fragmentation—resembling transient Okazaki fragments—was actually a consequence of Ung-mediated excision [10]. The physiological importance of dUTPase extends to human health, as mutations in the human *DUT* gene have been linked to severe monogenic syndromes characterized by diabetes, bone marrow failure, and elevated DNA replication stress, underscoring its indispensable role in human development and genome maintenance [11, 12]. While yeast-based genetic screening systems have established the mutagenic potential of uracil accumulation due to compromised dUTPase activity or unscheduled DNA synthesis [13, 14], the global genomic consequences of dUTPase deficiency and underlying mechanisms remain poorly defined. Specifically, how uracil repair intermediates orchestrate diverse mutational signatures, chromosomal rearrangements, and aneuploidy—and how these processes impact the stability of sensitive genomic features like the ribosomal DNA (rDNA) locus—has yet to be elucidated at a high-resolution, whole-genome scale.

In this study, we utilize *S. cerevisiae* to map the landscape of genomic instability induced by dUTPase deficiency. We demonstrate that Ung1-mediated BER acts as a primary driver to global point mutations to catastrophic DNA recombination. Chromosome loss is very common in low Dut1 cells, which is directly dependent on the presence of untreated uracil in DNA. We also uncover a specialized division of labor among translesion synthesis (TLS) polymerases and identify an adaptive contraction of repeated DNA regions as a response to chronic replication stress. Finally, we characterize the mechanics of “dUTPdysgenesis”, revealing how cross-parental enzymatic attack in the G1 phase triggers zygotic genome collapse. Our findings provide a comprehensive framework for understanding how metabolic crises hijack the DNA repair machinery to drive genomic instability.

## Materials and methods

### Yeast strains and medium

The diploid *S. cerevisiae* strain EG0 (wild-type) was generated by crossing the haploid *S. cerevisiae* strains SY165 (W303-1A background) [15] and YJSH1 (YJS329 background) [16]. To generate the DU strain for conditional *DUT1* expression, the endogenous *DUT1* promoter in both SY165 and YJSH1 was replaced with the *kanMX6-GAL1-3 × HA* cassette prior to mating. Detailed genotypes and construction protocols are provided in Supplementary Table S1 and the Supplementary Text, respectively. The primers used for strain construction are listed in Supplementary Table S2.

The DU strain was maintained in HG medium, consisting of 0.1% galactose (Macklin, D796635), 1% yeast extract (Oxoid, LP0021B), 2% peptone (Oxoid, LP0042B), and 3% raffinose (Aladdin, R111688) [17]. To restrict *DUT1* expression, the galactose concentration was reduced to 0.005% (LG medium). When required for selection, G418 (Macklin, G6021) and hygromycin B (Invitrogen, 10 687 010) were each added at a final concentration of 300 mg/l. HG his- met- medium contained 0.67% yeast nitrogen base (Shanghai Yuanye, S24483), 3% raffinose, 0.1% galactose, and 0.14% synthetic defined (SD) supplement lacking histidine and methionine. For screening white/red sectored colonies, HG-MAB6 medium was used, containing 0.67% yeast nitrogen base, 3% raffinose, 0.1% galactose, 0.001% adenine (Aladdin, A108804), 2% agar (SCRC, 10 000 561), and 0.14% SD supplement lacking arginine and adenine. The SD supplement was prepared as a dry amino acid/nucleobase mixture and added at a final concentration of 0.14%. The complete SD supplement included adenine, uracil, and a suite of essential amino acids (L-Trp, L-His, L-Arg, L-Met, L-Tyr, L-Leu, L-Ile, L-Lys, L-Phe, L-Glu, L-Asp, L-Val, L-Thr, and L-Ser); dropout supplements were prepared by omitting the specified components.

### Genetic manipulation

To delete *UNG1, APN1, REV1, REV3, RAD30, RAD51, RAD52, CHL1*, and *CTF18* targeted knockout cassettes were generated via PCR-based gene disruption using pUG72 [18] as the template. Yeast transformations were performed using the standard ssDNA/PEG/LiAc protocol [19]. Proper integration at the target loci and the subsequent loss of the coding sequences were confirmed by diagnostic genomic PCR. Diploid strains were generated by mating SY165 and YJS329-derived haploids, followed by selection on HG his- met- medium. To assess the extent of ssDNA exposure, EG0 and DU strains were transformed with a plasmid expressing the human *APOBEC3B* gene [20], yielding strains EG0-A3B and DU-A3B, respectively.

### Determination of cellular dNTPs using liquid chromatography–mass spectrometry (LC-MS)

Wild-type and DU strains were precultured overnight in 10 ml of HG medium at 30°C. To restrict *DUT1* expression, cells were inoculated into 10 ml of LG medium at an initial OD_600_ of 0.01. Cells were harvested upon reaching the mid-logarithmic phase (OD_600_ = 1.0) and immediately quenched in an extraction buffer containing 80% methanol and 0.1% formic acid that was precooled in −80°C freezer to arrest metabolism. Intracellular metabolites were extracted using a method described previously [21] with some modifications. Cell lysis was achieved via bead-beating and then followed with two rounds of centrifugation at 15 000 × *g* for 15 min at 4°C. The supernatant fraction containing soluble metabolites was harvested and dried using a CentriVap Concentrator system (Beijing JM Tech, China). Dried metabolite extracts were then re-suspended in 10 mM Ammonium acetate for injection. The levels of dNTPs were quantitatively analyzed in a negative ion mode using multiple reaction monitoring acquisition with a triple quadrupole mass spectrometer AB SCIEX Triple Quad 6500 + coupling to an Ultrahigh-performance liquid chromatography. Nucleotides were separated chromatographically on a HSS T3 column with polar embedded groups (ACQUITY UPLC HSS T3 Column, 100Å, 1.8 µm, 2.1 mm × 150 mm, Waters). Flow rate was set to 0.2 ml/min using a 17 min liquid chromatography (LC) method: Buffer A: 10 mM ammonium acetate (pH 8.0), Buffer B: 100% acetonitrile. 0–2 min, 1%–1% B; 2–10 min, 1%–8% B; 10–13.2 min, 8%–60% B; 13.2–13.3 min, 60%–90% B;13.3–16.3 min, 90%–90% B; 16.3–17 min, 90%–1% B. The retention time for each MRM peak was compared to an appropriate standard. The area under each peak was then quantitated using SCIEX OS 3.4 software and re-inspected for accuracy.

### Cell-cycle analysis by flow cytometry

Haploid strains SY165 and WDU (*pGAL1-DUT1* in the SY165 background) were cultured in 10 ml of HG medium to an OD_600_ of 0.5. For G1 phase synchronization, ∼10^8^ cells were harvested and treated with α-factor (final concentration 30 μg/ml) for 2.5 h at 30°C. Following synchronization, cells were washed twice with ice-cold sterile water and released into pre-warmed fresh medium. Samples were collected at 15-min intervals over a 3-h period. At each time point, cells were pelleted by centrifugation (12 000 revolutions per minute, 1 min), fixed in 70% ethanol, and incubated at room temperature for 30 min. Fixed cells were resuspended in 50 mM sodium acetate buffer (pH 7.5), followed by sequential treatment with RNase A (0.5 mg/ml at 37°C for 4 h) and Proteinase K (2 mg/ml at 50°C for 1 h). The samples were then stained with propidium iodide (25 μg/ml at 37°C for 1 h). DNA content was analyzed using a BD FACSCalibur system, acquiring at least 10 000 events per sample. Data processing was performed using FlowJo software.

### Mutation accumulation experiments

Mutation accumulation (MA) lines were established from single colonies and propagated through successive single-colony bottlenecks on the specified solid media. In each passage, one colony was randomly selected and restreaked onto a fresh plate to ensure the accumulation of mutations under minimal selective pressure. Specifically, MA lines for EG0 (50 independent isolates), DU (29 independent isolates), and their BER- or TLS-deficient derivatives (at least eight independent isolates for each mutant, detailed in Supplementary Table S3) were maintained on LG plates (0.005% or 0.02% galactose). Based on colony-forming unit assays [22] each passage was estimated to represent ∼25 cell divisions. Following the final passage, colonies were purified on HG medium prior to genomic DNA extraction and whole-genome sequencing. Mutation rates were calculated by dividing the total number of identified mutations by the product of the cumulative cell divisions and the diploid genome size (2.3 × 10^7^ bp).

### The next generation of whole-genome sequencing and variant calling

Genomic DNA of MA lines were extracted using the Apostle MiniGenomics kit according to the manufacturer’s instructions. Sequencing libraries were constructed using the VAHTS^®^ Universal Plus kit (Vazyme) and MGI Adapter Set 8, followed by circularization with the VAHTS^®^ Circularization Kit. Paired-end 150-bp sequencing was performed on the MGI DNBSEQ-T7 platform, yielding ∼1 Gb of raw data per sample. The average genomic coverage (50–70×) across all MA isolates was obtained to avoid depth-dependent detection bias. For data processing, raw reads were quality-assessed using FastQC (https://www.bioinformatics.babraham.ac.uk/projects/fastqc/) and trimmed for adapter sequences and low-quality bases (Phred score < 20) using Trimmomatic [23]. High-quality reads were aligned to the *S. cerevisiae* S288C reference genome (R64-1-1) using BWA-MEM [24], and the resulting alignments were processed and sorted with Samtools [25]. Single nucleotide variations (SNVs) and small insertions/deletions (InDels) were identified using VarScan (v2.3.9) based on Samtools mpileup files [26]. To ensure high-confidence variant calling, candidate mutations were filtered using the following stringent criteria: minimum read depth ≥20; variant-supporting reads ≥15; average base quality ≥20; and variant allele frequency ≥0.25. To eliminate false positives and background variants, all pre-existing parental single-nucleotide polymorphisms (SNPs) and variants detected in unsubcultured control strains were subtracted.

### Analysis of loss of heterozygosity, large deletions/duplications, and aneuploidy

Genome-wide loss of heterozygosity (LOH) and copy number variations were evaluated using a parental SNP-specific coverage pipeline. Based on informative SNPs between W303-1A and YJS329/YJM789, we defined the ratio of coverage (RC), where ∼0.5 represents heterozygosity. Interstitial (I-) and terminal (T-) LOH were distinguished by whether the LOH tract encompassed the telomere. Large-scale rearrangements (I-DUP, I-DEL, T-DUP, T-DEL) were called based on concurrent changes in total depth and SNP-specific RC (thresholds: > 0.7 for gains and < 0.05 for losses). Furthermore, whole-chromosome events were categorized as monosomy (one homolog RC < 0.05), trisomy (one homolog RC > 0.7), or uniparental disomy (UPD). UPD was specifically defined by the loss of one parental allele (RC < 0.05) coupled with the duplication of the corresponding homolog (RC > 0.7).

### Nanopore sequencing and analysis

Nanopore sequencing was performed as previously described [27]. Genomic DNA was extracted following the method of [28], with the modification that zymolyase was used instead of lyticase for yeast cell wall digestion. DNA samples were barcoded using the EXP-NBD104 kit and sequencing libraries were prepared with the SQK-LSK109 ligation kit (Oxford Nanopore Technologies, ONT). For each run, 700 ng of the pooled library was loaded onto a MinION flow cell (FLO-MIN106D R9.4.1, ONT) and sequenced on a MinION Mk1B device. Data acquisition and real-time base-calling were performed using MinKNOW (v21.02.1) with the integrated Guppy (v4.3.2) basecaller. The resulting long reads were aligned to the *S. cerevisiae* S288C reference genome (R64-1-1) using NGMLR [29]. Alignment files (.sam) were converted to sorted and indexed.bam files using Samtools [30]. The junctions of structural variants were called using Sniffles [29] under default parameters and formatted as .vcf files for downstream analysis. A structural variant was considered high-confidence only if supported by at least three independent split reads of ONT fully spanning the breakpoint junction.

### Western blotting

Yeast cells were grown overnight in the indicated media (10 ml) at 30°C and harvested for protein extraction. Whole-cell lysates were prepared using lysis buffer with glass bead disruption. Protein concentrations were measured using a bicinchoninic acid (BCA) assay, and 40 μg of total protein was loaded per lane. Proteins were resolved on 12% TGX gels and transferred onto polyvinylidene fluoride (PVDF) membranes using a Bio-Rad Trans-Blot Turbo system. Membranes were blocked for 1 h in Tris-buffered saline with Tween 20 (TBST) containing 5% nonfat dry milk and incubated with primary antibodies against HA-tagged Dut1 and GAPDH. The primary antibodies were anti-HA (Proteintech, 51064-2-AP, 1:1000) and anti-GAPDH (Proteintech, 10494-1-AP, 1:1000). After washing with TBST, membranes were incubated with horseradish peroxidase-conjugated goat anti-rabbit IgG secondary antibody (Servicebio, GB23303, 1:5000). Signals were detected by chemiluminescence.

### Rad52-YFP foci detection

To assess DNA damage and repair center assembly, Rad52-YFP foci were visualized in wild-type (YXM61) and *pGAL1-DUT1* (YXM61D) strains. Yeast cells were cultured in the specified media at 30°C until reaching mid-logarithmic phase (OD_600_ = 0.5). Live-cell imaging was performed using a high-resolution confocal microscope (Olympus FV3000, Japan). The frequency of focus formation was quantified by scoring cells harboring at least one distinct Rad52-YFP focus as positive. For each experimental condition, at least nine randomly selected fields containing a minimum of 350 total cells were analyzed. The percentage of Rad52-YFP-positive cells was calculated to represent the steady-state level of spontaneous DNA lesions.

### Pulsed-field gel electrophoresis

Yeast cells were cultured at 30°C for 24 h in the 25 ml HG or LG liquid medium with an initial OD_600_ of 0.05. Cells were harvested by centrifugation at 4000 × *g* for 5 min, and 100 mg of wet cell mass was collected. For each milligram of wet cell mass, 0.4 μl of lyticase solution (3 U/μl) and 9 μl of 1.0% low-melting-point agarose were added and thoroughly mixed. Aliquots (100 μl) of the resulting mixture were transferred into plug molds, and the plugs were removed after the agarose had solidified.

The plugs were incubated overnight at 30°C in buffer containing 500 mM ethylenediaminetetraacetic acid (EDTA) and 10 mM Tris (pH 7.5). They were then transferred to lysis buffer containing 500 mM EDTA (pH 7.5), 5% sodium *N*-lauroylsarcosinate, and 6 mg/ml proteinase K and incubated at 50°C for 24 h. After lysis, the plugs were rinsed once with ddH₂O and then washed for ∼3 days at 14°C with gentle agitation in buffer containing 1 mM EDTA and 2 mM Tris (pH 8.0). The washing buffer was replaced every 12 h until the plugs became transparent.

The processed plugs were loaded into an agarose gel, and chromosome-sized DNA molecules were separated using a CHEF Mapper XA System. Electrophoresis was performed in 0.5 × Tris–borate–EDTA (TBE) at 6 V/cm (200 V total), with switch times of 60–120 s and a field angle of 120°, for 28 h. After electrophoresis, the gel was stained for 30 min in an aqueous solution containing 0.5 μg/ml ethidium bromide and destained in ddH₂O for 30 min.

### Yeast red/white sectoring assay

Red/white sectoring assays were employed to identify reciprocal crossover (RCO) events and determine the timing of recombinational lesions. The diploid strain used for this analysis was homozygous for the *ade2-1* ochre mutation, which typically yields a red colony phenotype [31]. A single copy of the *SUP4-o* suppressor tRNA gene was integrated into the right arm of one chromosome IV homolog [31]. The presence of a single *SUP4-o* copy partially suppresses the *ade2-1* mutation, resulting in pink colonies [31]. An RCO occurring between the centromere and the *SUP4-o* locus leads to the segregation of daughter cells with either zero (red) or two (white) copies of *SUP4-o*, manifesting as red/white sectored colonies.

For the mating-based sectoring assay, WDUΔung1 cells were initially cultured in 10 ml of YPD medium for 6 h at 30°C to facilitate the accumulation of genomic uracil. Subsequently, SY166 cells (expressing *UNG1*) were added to the culture, and the mixture was incubated for 2 h to allow for mating and diploid formation. The cells were then plated on SD his- ura- selective medium and incubated at 30°C for 3 days. Red/white sectored colonies were identified and isolated for downstream analysis. Individual red and white sectors were separated, purified on selective media, and subjected to whole-genome sequencing.

### Statistical analysis

The distribution of mutation types was evaluated using Fisher’s exact test. Unless otherwise specified, differences in mutation rates and other rate-dependent metrics were compared via a two-sided Wilcoxon rank-sum test. Metabolomics data were analyzed using Welch’s *t*-test. Details of the statistical tests applied to specific experiments are provided in the corresponding figure legends.

## Results

### Experimental system design

To investigate the impact of dUTPase deficiency on genome stability, we constructed a conditional *DUT1* expression model in *S. cerevisiae*. The endogenous *DUT1* promoter was replaced with the *GAL1* promoter in two haploid backgrounds (SY165 [22] and YJSH1 [16]), which were mated to generate the diploid strain DU (Supplementary Table S1). SY165 is a standard laboratory strain with a W303-1A genetic background [22], while YJSH1 is a haploid strain with a well-defined genome sequence [16]. The two parental genomes harbor ∼55 000 SNPs uniformly distributed across all 16 chromosomes. This high density of heterozygous markers is essential for our study, as it enables high-resolution, genome-wide mapping of LOH events, as well as allele-specific copy-number alterations [32, 33]. On LG (0.005%) medium, Dut1 protein levels in DU were reduced to ∼30% of wild-type levels (Fig. 1B), resulting in significant growth inhibition (Fig. 1C). As expected, *DUT1* downregulation caused substantial changes in nucleotide concentrations (Supplementary Fig. S1). In DU cells, intracellular dUTP levels surged 128-fold, whereas pyrimidine metabolites downstream of dUTP hydrolysis, including dUMP and dTMP, were significantly reduced (Supplementary Fig. S1). Furthermore, the pools of dCTP and dGTP were also markedly diminished in these cells (Supplementary Fig. S1). Flow cytometry revealed a markedly prolonged S-phase in low Dut1 cells, indicating a substantial delay in DNA replication progression (Fig. 1D).

To accumulate genomic alterations, 29 DU-derived isolates were subcultured on LG plates for 10 (21 isolates) or 15 (8 isolates) cycles, alongside 50 wild-type control (EG0) isolates (32 for 60 cycles and 18 for 90 cycles). Following subculturing, colonies were purified on HG (0.1%) medium and subjected to whole-genome sequencing. This approach allowed for a comprehensive analysis of the rates and spectra of various genetic events, including SNVs, InDels, LOH, chromosomal rearrangements/structural variants (large deletions and duplications), and aneuploidy.

### Low expression of DUT1 stimulates global genome instability

#### Low DUT1 level led to a global elevation of DNA point mutations

Genomic analysis of the 29 DU-derived isolates identified 267 SNVs and 53 InDels (Supplementary Dataset S1). Based on a cumulative 8250 cell divisions of the 29 isolates, the mutation rates were estimated at 1.41 × 10^−9^ SNVs and 2.79 × 10^−10^ InDels per base per division (Fig. 2A and Supplementary Table S3). These rates represent a significant 4-fold and 9-fold elevation in SNVs and InDels frequencies, respectively, relative to wild-type levels (Fig. 2A; *P* <.001, Wilcoxon rank-sum test). Most InDels (50 out of 53) occurred within (AT)n microsatellites or homopolymeric A/T tracts (Supplementary Dataset S1).

**Figure 2.**
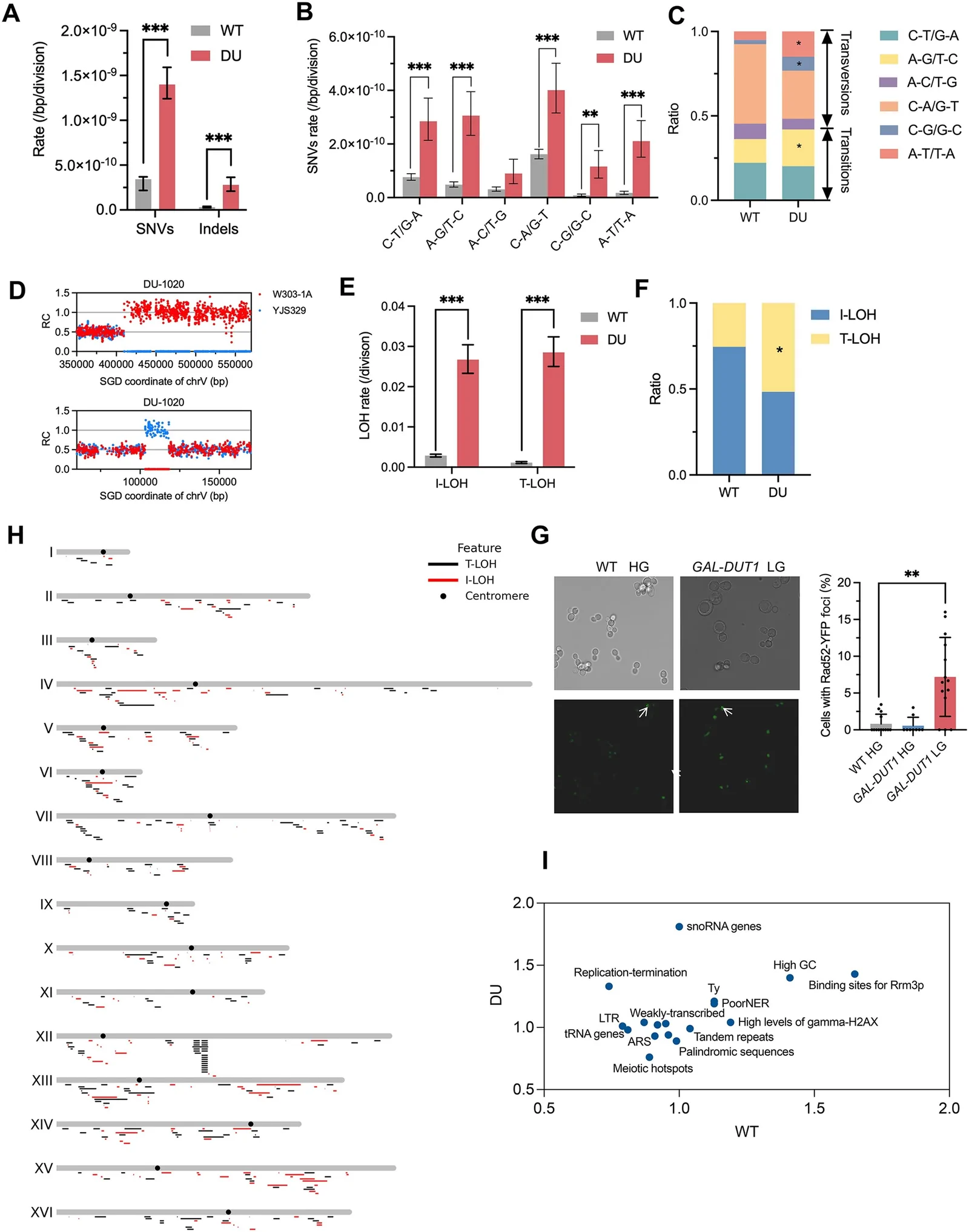
Reduced dUTPase expression promotes mutagenesis, LOH and recombination-associated genome instability. Unless otherwise indicated, WT denotes the wild-type diploid control EG0. In panel (H), WT denotes the Rad52-YFP reporter strain YXM61. (**A**) Genome-wide SNVs and Indels rates in WT and DU mutation-accumulation isolates. (**B**) Rates of individual SNVs classes, shown as complementary base-substitution pairs. (**C**) Relative contribution of the six base-substitution classes in WT and DU isolates. Substitutions are grouped as transitions and transversions. (**D**) Representative RC plots showing terminal LOH (T-LOH) and interstitial LOH (I-LOH) events. RC values near 0.5 indicate heterozygous regions, whereas values approaching 0 or 1 indicate loss of one parental allele. (**E**) Rates of I-LOH and T-LOH events in WT and DU isolates. (**F**) Relative ratios of I-LOH and T-LOH events in WT and DU isolates. (**G**) Genome-wide distribution of LOH tracts detected in DU mutation-accumulation isolates propagated under LG conditions. Chromosomes are ordered from I to XVI. Black and red segments indicate CO/break-induced replication (BIR)-associated and conversion-associated LOH events, respectively. (**H**) Representative bright-field and fluorescence images of Rad52-YFP foci in WT and *GAL-DUT1* cells grown under the indicated conditions. Arrows indicate Rad52-YFP foci. The right panel shows the percentage of cells with Rad52-YFP foci. Each point represents an independent microscopic field; bars indicate mean ± standard deviation. (**I**) Association between genomic features and LOH events in WT and DU isolates. Each point represents one genomic feature. Values above 1 indicate enrichment relative to the genomic background. Error bars in panels (A), (B), and (E) indicate 95% Poisson confidence intervals. Statistical significance is indicated as *P <.05, **P <.01, ***P <.001. *P*-values were calculated using the Wilcoxon rank-sum test for panels (A), (B), (E), and (H), and Fisher’s exact test for panels (C) and (F).

Notably, reduced dUTPase expression increased rates across all SNVs classes (Fig. 2B). The proportions of A-to-T/T-to-A (5%–15%), C-to-G/G-to-C (2.4%–8.2%), and A-to-G/T-to-C (14.1%–21.7%) substitutions were significantly elevated (*P* <.05, Fisher’s exact test) (Fig. 2C). Beyond frequency, low Dut1 imposed unique sequence-context preferences: T-A substitutions were highly biased toward 5′-CTT-3′ motifs, while T-C transitions favored 5′-CTG-3′ and 5′-TTG-3′ contexts (Supplementary Fig. S2). These results highlight that uracil-mediated genomic instability is a qualitative, sequence-dependent process, rather than a stochastic quantitative increase. Later we will discuss the mechanisms of such context-dependent mutation pattern.

#### Reduced dUTPase stimulates mitotic recombination

LOH is a frequent genetic event in diploids, categorized as either interstitial I-LOH or T-LOH based on whether the homologous tract is confined internally or extends to the telomere [22, 34, 35]. According to current mitotic recombination models, I-LOH is predominantly generated via the synthesis-dependent strand annealing (SDSA) pathway, which inherently favors noncrossover outcomes [36]. Conversely, T-LOH is primarily driven by either the resolution of double Holliday junctions (dHR) yielding crossovers or via the BIR pathway [36]. Representative profiles of an I-LOH and T-LOH in the subcultured isolates are illustrated in Fig. 2D. In the wild-type strain EG0, the spontaneous rates of I-LOH and T-LOH were 2.84 × 10^−3^ and 1.13 × 10^−3^ events per division, respectively (Fig. 2E and Supplementary Table S4). In contrast, analysis of 29 DU-derived isolates revealed 220 I-LOH and 235 T-LOH events, with rates significantly increasing to 2.67 × 10^−2^ and 2.85 × 10^−2^ events per division (Fig. 2E and Supplementary Table S4). These values represent 9.4-fold and 25.2-fold increases in I-LOH and T-LOH frequencies, respectively, compared to wild-type levels (*P* <.001, Wilcoxon rank-sum test). *DUT1*-low isolates exhibited a significantly higher T-LOH/I-LOH ratio than EG0 (Fig. 2F; *P* <.05, Fisher exact test), a signature consistent with patterns observed in yeast mutants with lower expression of replicating polymerases [17, 37]. The chromosome coordinates and patterns for all LOH events are detailed in Supplementary Datasets S2 and S3, respectively.

During mitotic growth, LOH events in diploid yeast are primarily driven by homologous recombination-mediated repair of DSBs [38]. Since the Rad52 protein coalesces at DSBs sites to form cytologically detectable foci [39], we quantified these foci to assess the prevalence of DNA damage. Compared to wild-type controls, *GAL-DUT1* cells cultured under LG conditions exhibited a 8.2-fold increase in the fraction of Rad52-YFP foci-positive cells (Fig. 2G). This significant elevation confirms that Dut1 limitation triggers a substantial accumulation of DSBs. To directly verify the physical accumulation of chromosomal DSBs, we performed Pulsed-field gel electrophoresis (PFGE) to assess whole-chromosome integrity. Consistent with the marked elevation in Rad52-YFP foci, chromosomes isolated from *DUT1*-limited cells (WDU) displayed prominent band intensity attenuation (particularly chromosome XII) compared to wild-type controls (Supplementary Fig. S3). This confirms that high-density uracil repair indeed triggers extensive DSBs *in vivo*. Additionally, deletion of *RAD51* or *RAD52* in the Dut1-deficient background exacerbated the growth defect compared to the low *DUT1* single mutant (Supplementary Fig. S4). This synthetic sickness further supports the notion that *DUT1* depletion induces recombinogenic DSBs that require repair via the homologous recombination pathway to maintain cellular viability.

To determine the spatial distribution of LOH events, we mapped all identified breakpoints across the 16 yeast chromosomes (Fig. 2H). Our results demonstrate that DNA recombination occurs genome-wide rather than being confined to specific loci (Fig. 2H). We assessed the overlap between LOH breakpoints and various genomic features using an enrichment analysis as described in [27, 37]. The analysis revealed that LOH breakpoints are mostly enriched in centromeres, high-GC regions, snoRNA genes, and Rrm3p binding sites (Fig. 2I and Supplementary Table S5). In contrast, most other genomic features showed no detectable association with recombination hotspots. These findings suggest that specific structural and protein-binding contexts preferentially trigger or facilitate the formation of recombinogenic lesions under dUTPase-deficient conditions.

#### Large-scale chromosomal rearrangements, copy-number variation at repetitive arrays, and aneuploidy

Across 29 DU-derived isolates, we identified 31 large structural rearrangements (>1 kb; Supplementary Dataset S4). Of the 31 events, 18 were classified as terminal deletions/duplications (T-Del/Dup; examples are shown in Fig. 3A) and 13 as interstitial events (I-Del/Dup). The rearrangement rate was calculated at 3.76 × 10^−3^ events per division, representing a 23-fold increase over wild-type levels (Fig. 3B; *P* <.01, Wilcoxon rank-sum test). Most breakpoints were flanked by Ty retrotransposons or long terminal repeat (LTR) sequences (Supplementary Dataset S4), suggesting that these events are primarily driven by nonallelic homologous recombination between repetitive elements. The T-Del/Dup events frequently occurred in pairs within a single isolate, a pattern consistent with translocations between nonhomologous chromosomes [27, 33]. In contrast, I-Del/Dup events likely arise from unequal crossovers between sister chromatids [27, 33]. To definitively resolve these structures, we performed Nanopore sequencing on three DU isolates (DU-1031, DU-1032, and DU-1037). This analysis confirmed the junction sequences of three rearrangement events (Fig. 3C), providing direct evidence for translocation between nonhomologs.

**Figure 3.**
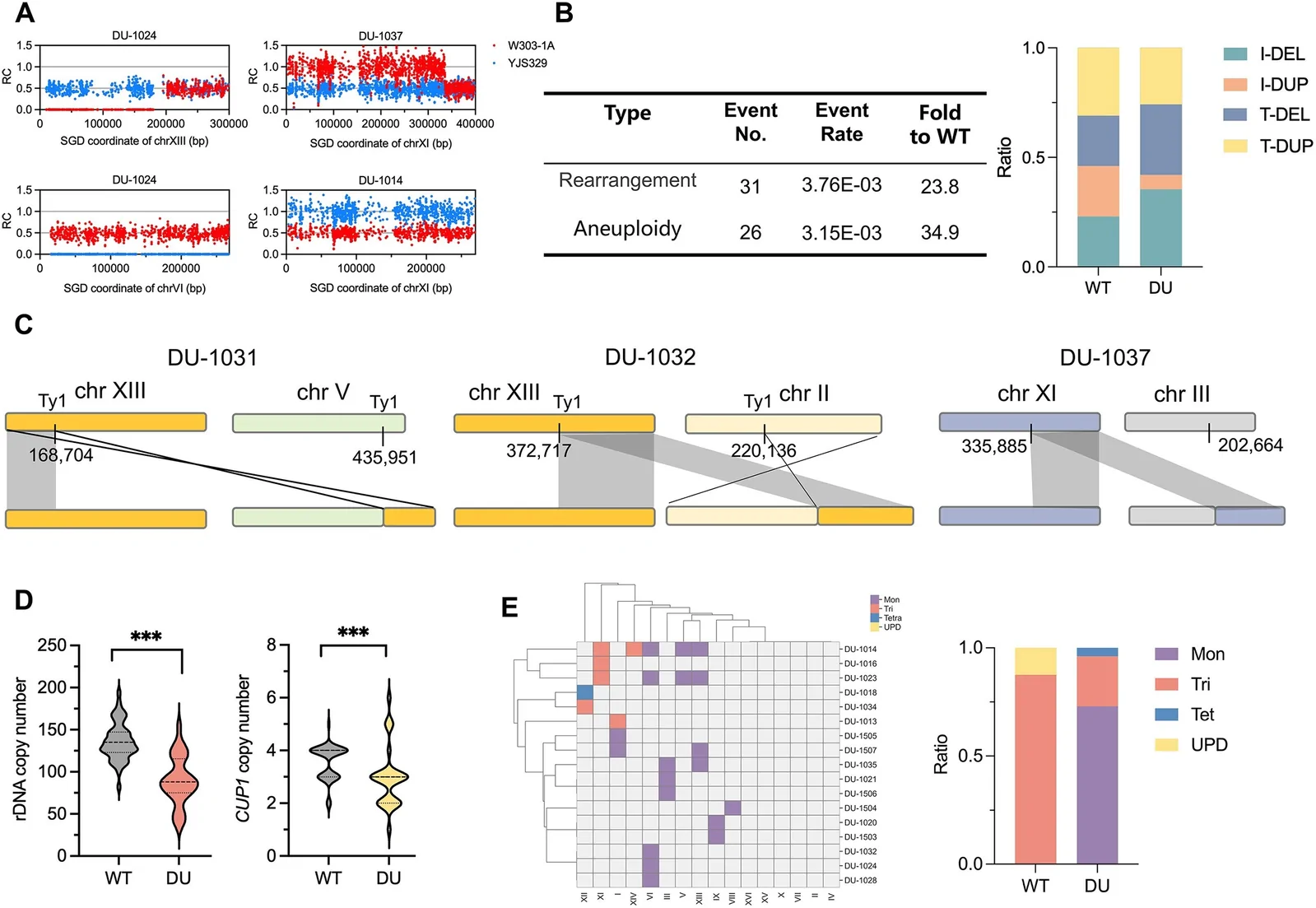
Reduced dUTPase expression promotes large-scale chromosomal rearrangements and aneuploidy. WT denotes the wild-type diploid control EG0. (**A**) Relative RC plots showing large-scale copy-number alterations in DU isolates based on parental SNP-specific read depth. RC values near 0.5 indicate the diploid baseline, whereas segmental losses or gains shift the signal downward or upward, respectively. Examples of terminal deletion (T-DEL), terminal duplication (T-DUP), monosomy (Mon), and trisomy (Tri) are shown. (**B**) Summary of rearrangement and aneuploidy events detected in DU mutation-accumulation isolates. Event rates are shown per cell division, with fold changes relative to WT. The right panel shows the relative ratios of interstitial deletions (I-DEL), interstitial duplications (I-DUP), T-DEL, and T-DUP events in WT and DU isolates. (**C**) Nanopore sequencing verified the breakpoints and junctions of chromosomal rearrangements in three DU isolates. Colored bars indicate the involved chromosomes, and gray shaded regions indicate duplicated or rearranged chromosomal segments. Black lines connect inferred breakpoint junctions between chromosome arms. (**D**) Estimated rDNA and *CUP1* copy numbers in WT and DU isolates inferred from sequencing coverage. Dashed lines indicate median values. (**E**) Aneuploidy profiles of DU isolates. The heatmap shows chromosome-level copy-number changes across individual DU isolates, and the right panel shows the relative ratios of Mon, Tri, tetrasomy (Tet/Tetra) and UPD events in WT and DU isolates. Statistical significance is indicated as ***P <.001. *P*-values in panel (C) were calculated using the Wilcoxon rank-sum test. Panels (B) and (D) were analyzed using Fisher’s exact test.

In *S. cerevisiae*, the rDNA locus consists of tandem repeats on chromosome XII, with an average of 136 copies per haploid genome in wild-type strain EG0 (Fig. 3D). Under Dut1-limited conditions, however, the rDNA copy number significantly decreased to an average of 90 (Fig. 3D; *P* <.001, Wilcoxon rank-sum test). A similar contraction was observed at the *CUP1* locus, another tandemly repeated region (Fig. 3D; *P* <.001, Wilcoxon rank-sum test). These findings indicate a general propensity for copy number loss within repetitive genomic regions in dUTPase-deficient cells. This trend mirrors our observation of a higher frequency of I-Del compared to I-Dup (Fig. 3B).

Aneuploidy was frequently observed in Dut1-deficient strains, with a rate of 3.15 × 10^−3^ events per cell division—a 35-fold increase over wild-type levels (Fig. 3B; *P* <.001, Wilcoxon rank-sum test). While wild-type controls (50 isolates) exhibited seven trisomies and one UPD, the DU isolates were characterized predominantly by monosomy (2n-1) (19 out of 26 events; Fig. 3E and Supplementary Dataset S5). Furthermore, combining *DUT1* depletion with the loss of *CTF18* (a PCNA clamp-loading and cohesion establishment factor) or *CHL1* (a DNA helicase essential for cohesion establishment) resulted in pronounced synthetic lethality (Supplementary Fig. S4). These negative genetic interactions highlight the indispensable role of sister chromatid cohesion in tolerating uracil-induced replication stress and aneuploidy.

### Ung1-mediated BER contributed to genome instability in low Dut1 cells

Given that DNA uracil is primarily processed via the Ung1-mediated BER pathway (Fig. 4A) [6], we investigated the requirement of Ung1 for *DUT1*-limited mutagenesis. In the *GAL1-DUT1 ung1∆* double mutant, the overall SNVs rate exhibited a slight, statistically nonsignificant decline (Fig. 4B, Supplementary Table S3, and Supplementary Dataset  S6). However, the mutational spectrum underwent a profound shift: C-to-T/G-to-A transitions surged 2.4-fold, while all other substitution classes were markedly suppressed to the wild-type level (Fig. 4C). These data indicate a dual role for Ung1: its absence precludes the repair of spontaneous cytosine deamination—thereby elevating C-to-T transitions—while simultaneously abrogating the majority of other mutations typically triggered by dUTPase deficiency. This suppression likely stems from the reduction of mutagenic AP sites, which are generated upon Ung1-mediated uracil excision (Fig. 4A). To verify the mutagenic potential of these intermediates, we analyzed genomic mutations in the *GAL1-DUT1 apn1∆* double mutant. Due to the severe growth defect of this strain under standard LG (0.005%) conditions, we conducted the MA assay at 0.02% galactose concentration, using the *GAL1-DUT1* (DU^a^) strain as a parallel control under identical conditions. The *GAL1-DUT1 apn1∆* mutant exhibited a 21-fold higher SNVs frequency compared to the control, confirming the potent mutagenicity of AP sites (Fig. 4D and Supplementary Table S3; *P* <.001, Wilcoxon rank-sum test). Intriguingly, the mutational spectrum of the *GAL1-DUT1 apn1∆* strain was heavily biased toward thymine positions (T-to-A, T-to-C, and T-to-G), which increased 7.5-, 29.7-, and >100-fold, respectively (Supplementary Fig. S5). This thymine-centric signature contrasts sharply with the global elevation of all SNVs classes observed in *DUT1*-low strains under standard LG conditions. To further complement these observations, we constructed an *APN1*-overexpressing strain in the DU background (DUU-APN1). Compared to the DU control, DUU-APN1 exhibited a noticeable reduction in T-to-A (65.8%) and T-to-G (82.2%) transversion frequencies, whereas C-to-A and C-to-G substitutions were elevated by 150% and 78%, respectively (Supplementary Fig. S6A; see the ‘Discussion’ section for mechanistic details). These results demonstrate that while augmenting AP site processing capacity successfully mitigates mutations at thymine positions (where uracil is primarily misincorporated), it concurrently introduces nonthymine mutations elsewhere in the genome. Collectively, these results suggest that while mutagenesis in *DUT1*-deficient cells depends on the initiation of BER by Ung1, the resulting SNVs are partially driven by the direct bypass of static AP sites.

**Figure 4.**
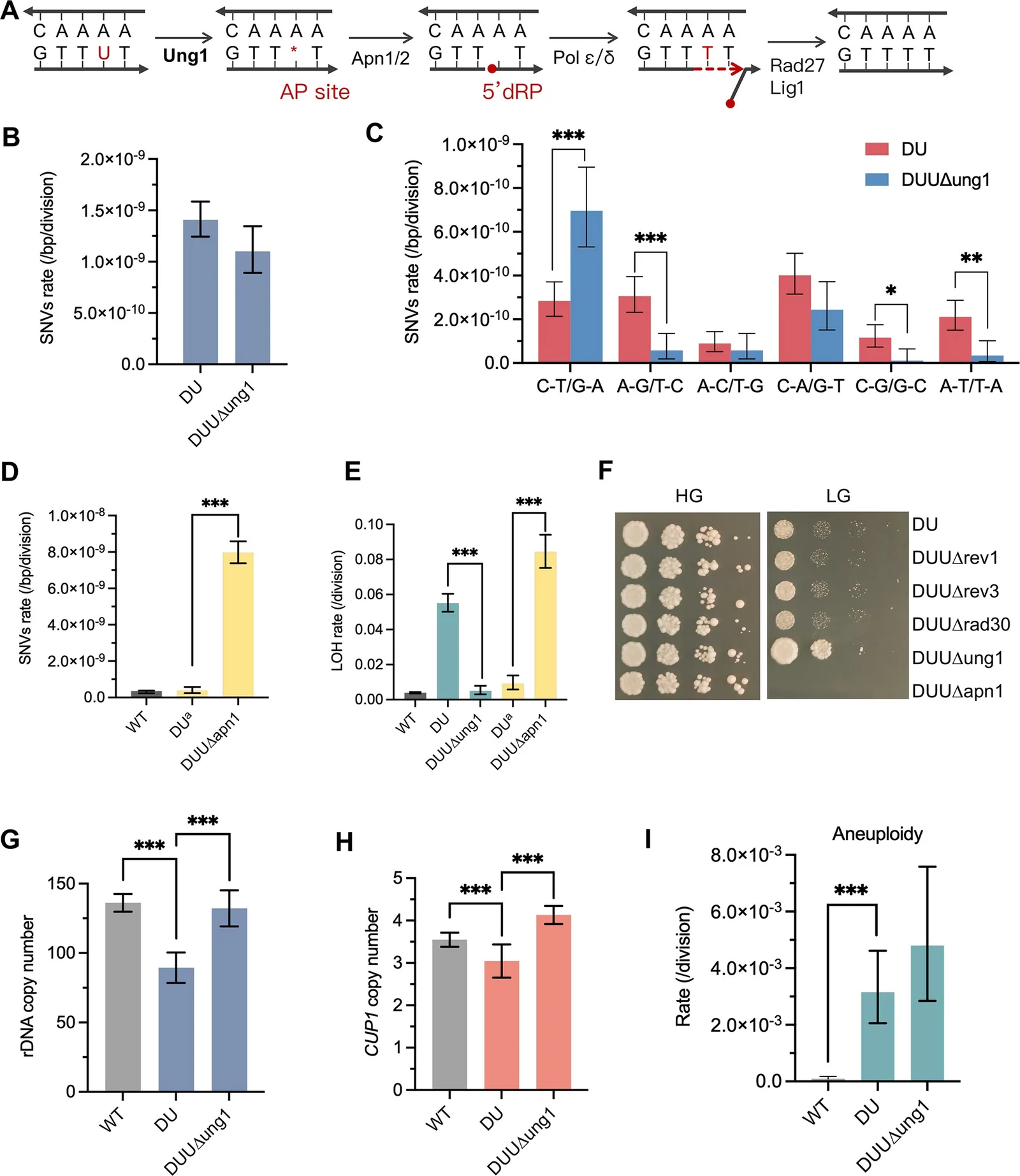
Uracil excision by BER contributes to genome instability under reduced dUTPase expression. WT denotes the wild-type diploid control EG0. (**A**) Schematic of uracil-initiated BER. Ung1 removes uracil from DNA to generate an AP site, which is further processed by Apn1, DNA polymerases and ligase to complete repair. (**B**) Genome-wide SNVs rates in DU and DUUΔung1 mutation-accumulation isolates. (**C**) Rates of individual SNV classes in DU and DUUΔung1 isolates, shown as complementary base-substitution pairs. (**D**) LOH rates in WT, DU, and BER-deficient derivatives. (**E**) Rates of large-scale chromosomal rearrangements in WT, DU and DUUΔung1 isolates. (**F**) Growth phenotypes of DU and derivative strains carrying deletions in TLS genes (*REV1, REV3* and *RAD30*) or BER genes (*UNG1* and *APN1*) under HG and LG conditions. (**G**) Estimated rDNA copy numbers in WT, DU and DUUΔung1 isolates inferred from sequencing coverage. (**H**) Estimated *CUP1* copy numbers in WT, DU and DUUΔung1 isolates inferred from sequencing coverage. (**I**) Aneuploidy rates in WT, DU, and DUUΔung1 isolates. Error bars in panels (B), (C), and (E)–(I) indicate 95% Poisson confidence intervals. Statistical significance is indicated as *P <.05, **P <.01, ***P <.001. *P*-values were calculated using the Wilcoxon rank-sum test for panels (B), (C), and (E)–(I).

Beyond SNVs, we observed that the InDels rate in the *DUT1*-limited strain was attenuated by 79% upon *UNG1* deletion (Supplementary Fig. S7 and Supplementary Table S3; *P* <.01, Wilcoxon rank-sum test). This dramatic reduction indicates that InDels formation is largely contingent on Ung1-mediated initiation of the BER. Furthermore, the loss of *APN1* led to a 4.5-fold increase in the InDels rate in *DUT1*-deficient cells (Supplementary Fig. S7 and Supplementary Table S3; *P* <.05, Wilcoxon rank-sum test). However, the 4.5-fold increase in the InDels rate upon *APN1* deletion was substantially lower than the 9-fold surge observed in *DUT1*-limited cells relative to wild-type levels. This quantitative discrepancy implies that while static AP sites contribute significantly to mutagenesis—likely via DNA polymerase slippage—the subsequent enzymatic processing of these intermediates may also act as a crucial inducer of InDels events.

We investigated whether the elevated recombination rates in *DUT1*-limited cells were dependent on the processing of genomic uracil. Notably, deletion of *UNG1* suppressed the LOH rate by 91% in the DU strain, reducing it to levels comparable to wild-type (Fig. 4E and Supplementary Table S4). Furthermore, the copy loss at rDNA (Fig. 4G) and *CUP1* (Fig. 4H) repeats was markedly suppressed upon *UNG1* deletion, confirming that uracil-initiated BER processing—rather than the uracil base itself—drives most recombinogenic damage in *DUT1*-deficient cells. In contrast, we observed a 9.3-fold elevation of LOH frequency in the *GAL1-DUT1 apn1*Δ double mutant compared to *GAL1-DUT1* (DU) (Fig. 4E). Consistent with these trends, the deletion of *UNG1* improved the growth of *DUT1*-limited cells, whereas the loss of *APN1* severely exacerbated growth inhibition (Fig. 4F). These findings demonstrate that AP sites are significantly more cytotoxic and recombinogenic than the uracil base per se. Consistently, PFGE analysis confirmed that *APN1* deletion DSBs formation in *DUT1*-limited cells (Supplementary Fig. S3). Intriguingly, the *GAL1-DUT1 apn1*Δ strain exhibited a distinctive recombination landscape, with I-LOH accounting for 78% of events, a stark contrast to the 48% observed in *GAL1-DUT1* (Supplementary Table S4). This dramatic shift in LOH patterns suggests that the downstream processing of AP sites (likely generating single-strand gaps), rather than the static AP sites themselves, serves as the primary driver of the massive induction of recombination in *DUT1*-deficient cells. This hypothesis was substantiated by evaluating the *APN1*-overexpressing strain. Overexpression of *APN1* marginally increased the overall frequencies of LOH (1.1-fold) and rearrangements (1.4-fold) (Supplementary Fig. S6B; Wilcoxon rank-sum test, *P* >.05) but reduced the relative proportion of I-LOH to 34%, less than half of that observed in the *APN1* deletion mutant (Fisher exact test, *P* <.05). This pivotal finding confirms that the enzymatic processing of AP sites actively provokes homologous recombination, predominantly channeling the repair outcomes toward T-LOH.

In contrast to DNA mutations and recombination, the fact that *UNG1* deletion failed to suppress aneuploidy—and even led to a 52% increase, albeit without reaching statistical significance (Fig. 4I)—highlights that numerical chromosomal instability in this context is mainly governed by a distinct mechanism independent of uracil excision. Interestingly, we found that *APN1* overexpression further elevated the frequency of chromosomal aberrations by 60%. Together, these results indicate that both the persistence of intact uracil bases and the downstream processing of AP sites exert a more profound detrimental impact on chromosome segregation fidelity.

### The complex role of TLS in *DUT1*-limited mutagenesis

TLS employs error-prone DNA polymerases to bypass DNA lesions, ensuring the completion of replication [40–42]. In yeast, this process involves DNA polymerases Rev1, Pol ζ, and Pol η (Rad30) [43–45]. To determine how TLS DNA polymerases shape the mutational landscape under dUTPase deficiency, we characterized *DUT1*-limited strains lacking *REV1, REV3*, or *RAD30*. Despite maintaining growth rates comparable to the DU parental strain on LG medium (Fig. 4F), these TLS-deficient isolates exhibited a sophisticated functional partitioning in their mutational signatures, suggesting distinct roles for each polymerase in bypassing uracil-derived lesions.

*REV1* and *REV3* absence led to a 33% and 18% reduction in the overall SNVs rate, respectively (Fig. 5A). Specifically, C-to-G/G-to-C transversions were virtually abolished in both the *rev1∆* and *rev3∆* mutants (Fig. 5B). However, distinct mutational profiles emerged for each subunit: *rev3∆* significantly decreased A-to-G/T-to-C transitions by 40%, while *rev1∆* eliminated A-to-C/T-to-G transversions by 85% (Fig. 5B). In contrast, *RAD30* deletion significantly increased the total SNVs rate, particularly for A-to-C/T-to-G and C-to-A/G-to-T substitutions, which rose by 198% and 74%, respectively (Fig. 5B). Furthermore, *REV1* and *REV3* deletions significantly elevated InDels frequencies, whereas the effect of *RAD30* was moderate (Fig. 5C). These results suggest a specialized division of labor among TLS polymerases: while Pol η facilitates relatively high-fidelity bypass of certain dUTPase-induced lesions, the Rev1–Pol ζ pathway serves as a driver of SNVs formation but protects against InDels formation.

**Figure 5.**
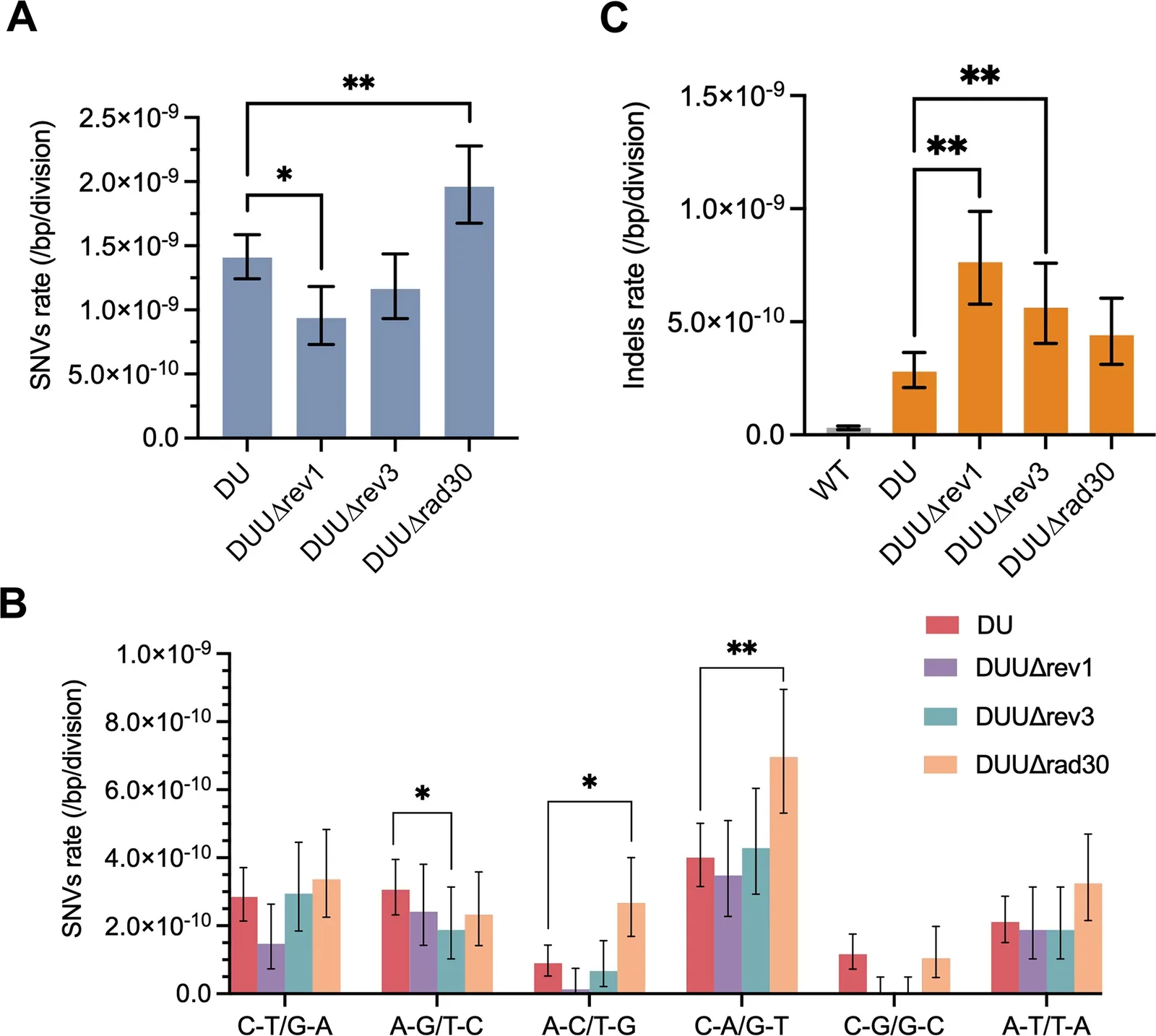
TLS DNA polymerases modulate mutation formation under reduced dUTPase expression. (**A**) Genome-wide SNV rates in DU and TLS-deficient derivatives. (**B**) Rates of individual SNV classes in DU and TLS-deficient derivatives, shown as complementary base-substitution pairs. (**C**) Genome-wide indel rates in WT (EG0), DU, and TLS-deficient derivatives. Error bars in panels (A)–(C) indicate 95% Poisson confidence intervals. Statistical significance is indicated as *P <.05, **P <.01, ***P <.001. *P*-values were calculated using the Wilcoxon rank-sum test.

### Analysis of the timing of recombinational DNA lesions in Dut1-deficient yeast

To determine the specific cell-cycle stage during which dUTPase-induced DSBs arise, we utilized a sector-based genetic assay (Fig. 6A). A *GAL1-DUT1* diploid (WYDU) was generated by crossing WDU and YDU (Supplementary Table S1). The haploid strain YDU carries a YJM789 genetic background, which is widely crossed with W303-1A-derived strains to generate hybrid diploids for high-resolution LOH detection [32, 33]. Additionally, the distal right arm of chromosome IV in YDU harbors an integrated *SUP4-o* suppressor tRNA gene, which suppresses the homozygous *ade2-1* ochre mutation. Consequently, in the diploid strain WYDU, RCOs occurring between the centromere and the *SUP4-o* locus lead to the segregation of daughter cells containing zero or two copies of *SUP4-o*, manifesting as distinct red/white sectored colonies. This system allows for the high-resolution reconstruction of recombination events: a DSB occurring in S or G2 phase involves only one sister chromatid and is characterized by a 3:1 gene conversion tract. In contrast, a G1-initiated DSB, when replicated, leads to breaks on both sister chromatids, typically resulting in a 4:0 or even more complicated conversion pattern.

**Figure 6.**
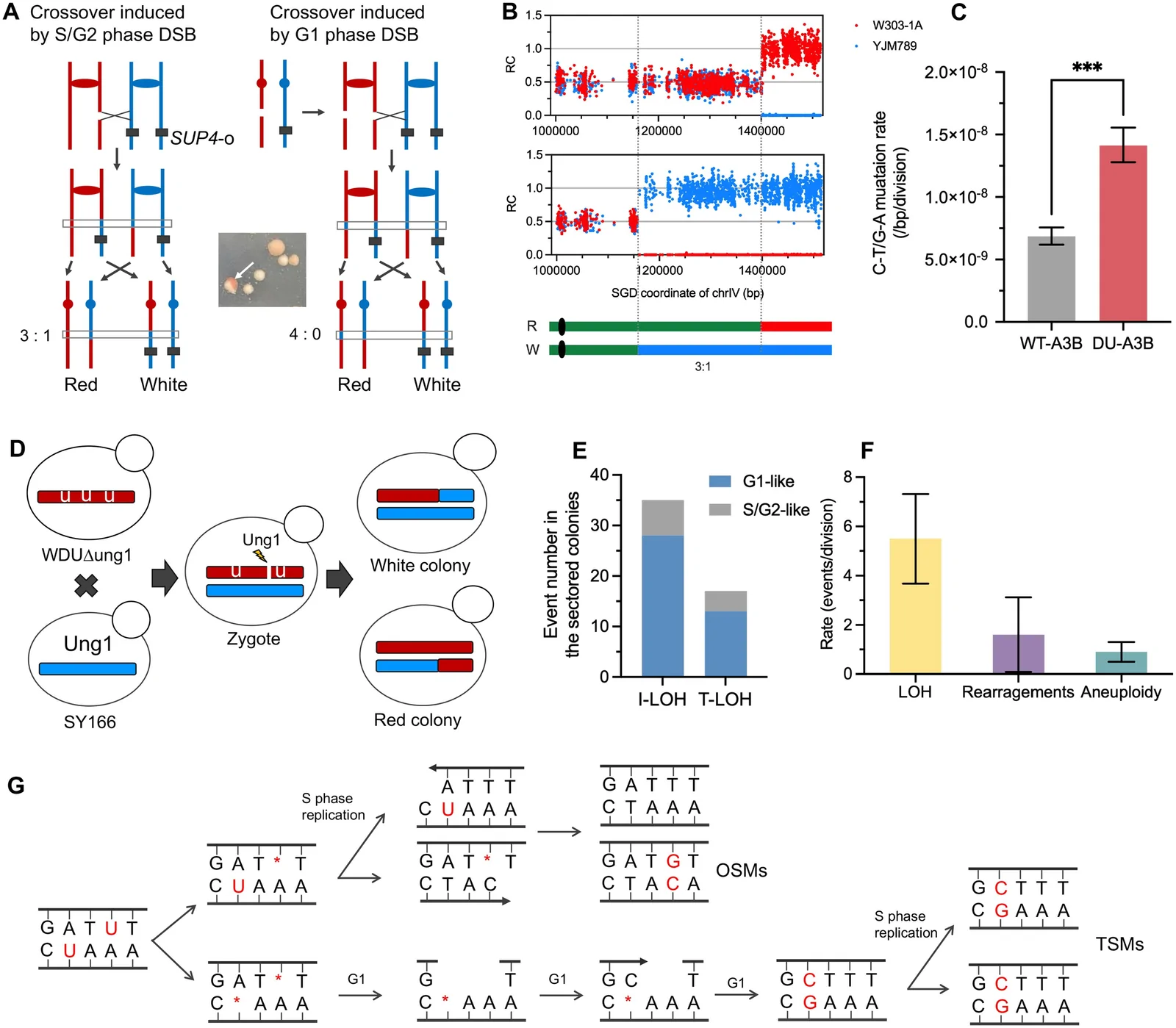
Sectoring-based analysis uncovers the timing of dUTPase deficiency-induced recombinational lesions. (**A**) Schematic of the red/white sectoring assay used to infer the timing of RCO events on chromosome IV. The W303-1A homolog is shown in red and the YJM789-derived homolog is shown in blue. The YJM789 homolog carries *SUP4*-o, and both homologs carry *ade2-1*. Diploids with zero, one, or two copies of *SUP4*-o form red, pink, or white colonies, respectively. Crossover-associated conversion tracts are shown as boxed regions. A 3:1 conversion pattern is consistent with an S/G2-like event, whereas a 4:0 pattern is consistent with a G1-like event. (**B**) RC plots showing crossover-associated gene conversion patterns in the red and white sectors. RC values near 0.5 indicate heterozygous regions, whereas values approaching 0 or 1 indicate loss of one parental allele. The lower schematic summarizes the corresponding 3:1 segregation pattern between the red and white sectors. (**C**) C-to-T/G-to-A mutation rates in WT and DU strains expressing APOBEC3B. (**D**) Experimental design for detecting recombination events generated after mating WDUΔung1 cells with *UNG1*-expressing SY166 cells. Uracil accumulation in the WDUΔung1 parent is exposed to Ung1 after zygote formation, allowing the timing and direction of repair-associated RCO events to be analyzed in sectored colonies. (**E**) Numbers of I-LOH and T-LOH events detected in 10 selected sectored colonies derived from zygotes, classified as G1-like or S/G2-like according to their segregation patterns. (**F**) The rates of LOH, chromosomal rearrangements, and aneuploidy detected in the 10 selected pairs of sectored colonies. (**G**) Model for the formation of one-strand mutations (OSMs) and TSMs after uracil excision. Uracil incorporation followed by Ung1-mediated excision generates AP sites. If an AP site is bypassed or repaired on only one DNA strand, the resulting lesion can be fixed after S-phase replication as a OSM. In contrast, when AP sites or repair intermediates occur on both complementary strands before replication, gap filling and repair synthesis may generate coordinated mutations on the two strands, producing two-strand mutations (TSMs). Red letters indicate newly formed mutations, and red asterisks indicate AP sites. Error bars in panels (C) and (F) indicate 95% Poisson confidence intervals. Statistical significance is indicated as ***P <.001. *P*-values were calculated using the Wilcoxon rank-sum test.

Following a 6-hour incubation in YPD to deplete the Dut1 protein, WYDU cells exhibited a red/white sectoring frequency of 3.2 × 10^−3^ (35/10 874), representing a substantial ∼91-fold elevation over wild-type levels. Whole-genome sequencing of 20 independent sectored colonies revealed that eight RCO events on the right arm of chromosome IV exhibited a 3:1 conversion pattern (one example is shown in Fig. 6B), while the 11 displayed 4:0 or hybrid patterns (Supplementary Dataset S7). These data indicate that 42% of chromosomal crossovers in low Dut1 cells arise from lesions initiated during the S/G2 phase.

### Increased ssDNA exposure in dUTPase-deficient cells

Abundant AP sites are expected to exacerbate SSBs, potentially increasing the exposure of ssDNA. To test this hypothesis, we expressed the cytidine deaminase APOBEC3B in wild-type (WT-A3B) and *DUT1*-limited (DU-A3B) strains. Since APOBEC3B preferentially deaminates cytosines within the 5′-TC-3′ motif exclusively on ssDNA [46], the resulting C-to-T mutation rate serves as a sensitive proxy for ssDNA prevalence during replication [47]. Our genomic analysis revealed that DU cells exhibited a 2.4-fold higher rate of C-to-T mutations at 5′-TC-3′ sites compared to wild-type controls (Fig. 6C). This confirms that reduced dUTPase levels promote persistent ssDNA exposure.

### dUTPdysgenesis: Ung1-mediated genomic instability in incompatible crosses

We previously described a hybrid incompatibility in *S. cerevisiae* where RNase H2-mediated incision of ribonucleotide-containing genomes triggers a dysgenic cross [48]. Here, we report dUTPdysgenesis, induced by high-density dUTP incorporation. In this experiment, we mated a dUTP-loaded haploid (WDUΔung1; *GAL1-DUT1* and *ung1*Δ in the W303-1A background) with a wild-type haploid SY166 (YJM789 background) (Fig. 6D). Hybrids were subsequently recovered on selective media to assess the impact of cross-parental uracil excision on zygotic genome stability. The resulting zygotes exhibited a red/white sector frequency of 7.16 × 10^−4^—a 20-fold increase over spontaneous rates—indicating massive DNA recombination immediately following fertilization. Whole-genome sequencing of 10 zygote-derived sectored colonies identified 55 LOH events (10 were selected RCOs on chromosome IV). After excluding two BIR and one noninformatic complex I-LOH, we analyzed the remaining 52 events (35 I-LOH and 17 T-LOH) to determine the timing of damage and template preference (Supplementary Dataset S8). Notably, 41 of these 52 events (79%) exhibited 4:0 containing gene conversion patterns (Fig. 6E), demonstrating that most recombinogenic lesions are initiated in the G1 phase prior to the first round of DNA replication. Furthermore, in 49 of these 52 events, the wild-type (dUTP-free) homolog served as the repair template (Supplementary Dataset S8). These findings suggest that upon mating, Ung1 provided by the wild-type parent immediately excises uracil from the WDUΔung1 genome, triggering DSBs in G1. Our analysis indicates that these 55 LOH events should occur primarily between zygote formation and the first cell division, representing a rate of 5.5 events per division (Fig. 6F)—a 1385-fold increase over spontaneous levels. Similarly, we observed frequent large-scale amplifications and deletions (1.6 events per division) (Fig. 6F), occurring at a frequency 7216-fold higher than spontaneous rates. This paternal-style genomic attack prior to replication drives catastrophic chromosomal shattering, resulting in a three-order-of-magnitude increase in LOH and structural instability in the progeny.

In addition to the observed genomic instability, dUTPdysgenesis induced severe chromosomal segregation errors, including two UPD and seven monosomy events across 10 sector pairs—a rate of ∼0.9 events per division that marks a four-order-of-magnitude elevation above baseline spontaneous levels (Fig. 6F). Notably, these segregation defects were not randomly distributed but were heavily biased toward the uracil-preloaded W303-1A homologs (six events versus one in YJM789). This nonrandom loss of homologs provides compelling evidence that uracil residues compromise the segregation machinery in *cis*. It suggests that the physical or chemical alterations imposed by uracil on specific DNA strands—rather than a systemic cellular stress (*trans*-effect)—are the primary drivers of this massive chromosomal instability.

Among the 10 pairs of sequenced sectored colonies, we identified 58 SNVs (Supplementary Dataset S9). These were categorized into two groups: 30 TSMs, which were present at the same genomic site in both daughter cells, and 28 OSMs, which were restricted to a single daughter cell (Supplementary Dataset S9). The presence of TSMs implies that these mutations were fixed on one of the homologous chromosomes prior to the first round of DNA replication (Fig. 6G). Notably, both TSMs and OSMs exhibited highly consistent mutational signatures, with over 78% of substitutions occurring at T/A sites, and T-to-G/A-to-C being the most frequent class (Supplementary Dataset S9). This specific spectrum allows us to delineate the timing of these events. Had these SNVs occurred prior to mating, they would be predominantly localized to the WDUΔung1 homolog due to its significantly higher mutation rate. However, the mutational profile of the DUUΔung1 strain is dominated by C-to-T/G-to-A substitutions (63%; Fig. 4C), which is divergent from the T/A-biased signature observed in TSMs. Conversely, if these SNVs were generated after the first zygotic division, they would manifest exclusively as OSMs and, mirroring the spontaneous mutations seen in wild-type strains, occur primarily at C/G sites [22]. Consequently, we conclude that these 58 SNVs were predominantly generated during the window between zygote formation and the completion of the first round of DNA replication. The mechanistic basis for these events, particularly the high frequency of TSMs, is addressed further in the ‘Discussion’ section.

## Discussion

### Genetic mechanisms underlying the point mutations in dUTPase deficient cells

Our study reveals that uracil overload drives a global elevation across all base substitution types, rather than being restricted to thymine positions (Fig. 2B). Notably, deleting *UNG1* in *DUT1* low-expression strains provoked a surge in C-to-T transitions while significantly suppressing all other SNV classes (Fig. 4C). This confirms that the mutagenic effect of uracil overload is fundamentally dependent on Ung1-mediated BER. Previous studies using yeast reporter gene assays have suggested that SNVs resulting from Dut1 functional defects are primarily induced by AP sites [14]. However, despite the dramatic increase in SNVs frequency upon *APN1* deletion (Fig. 4D), our genome-wide data indicate that direct bypass of static AP sites is not the primary driver of mutagenesis in *DUT1*-deficient cells. In *GAL1-DUT1 apn1*Δ strains, mutations predominantly occur at thymine positions as T-to-G transversions (Supplementary Fig. S3), a signature distinct from the global elevation observed in *DUT1*-low MA strains (Fig. 2B). Correspondingly, overexpression of *APN1* (DUU-APN1) reduced T-to-G transversions but concurrently elevated mutations at nonthymine positions (Supplementary Fig. S6A), further demonstrating that both the direct bypass of AP sites and subsequent downstream processing steps contribute to mutagenesis in *DUT1*-deficient cells.

We propose that SNVs mutagenesis in *DUT1*-low cells is driven by multiple distinct mechanisms. First, TLS DNA polymerases induce base mismatches during the direct bypass of static AP sites generated by Ung1-mediated uracil excision (Fig. 7A). Our results showed that the deletion of *REV1* reduced T-to-G transversions by 85% (Fig. 5B). Given the established dCMP transferase activity of Rev1, we conclude that T-to-G mutations in *DUT1*-low strains result from Rev1-mediated incorporation of C opposite uracil-derived AP sites (Fig. 7A). In contrast, T-to-C transitions were significantly reduced in the absence of *REV3* (Fig. 5B), suggesting that Rev3 may incorporate G opposite these AP sites (Fig. 7A). The suppression of T-to-G mutations by Rad30 suggests that it can accurately incorporate A opposite uracil-derived AP sites (Fig. 7A), thereby preventing the mutagenic C-incorporation by Rev1.

**Figure 7.**
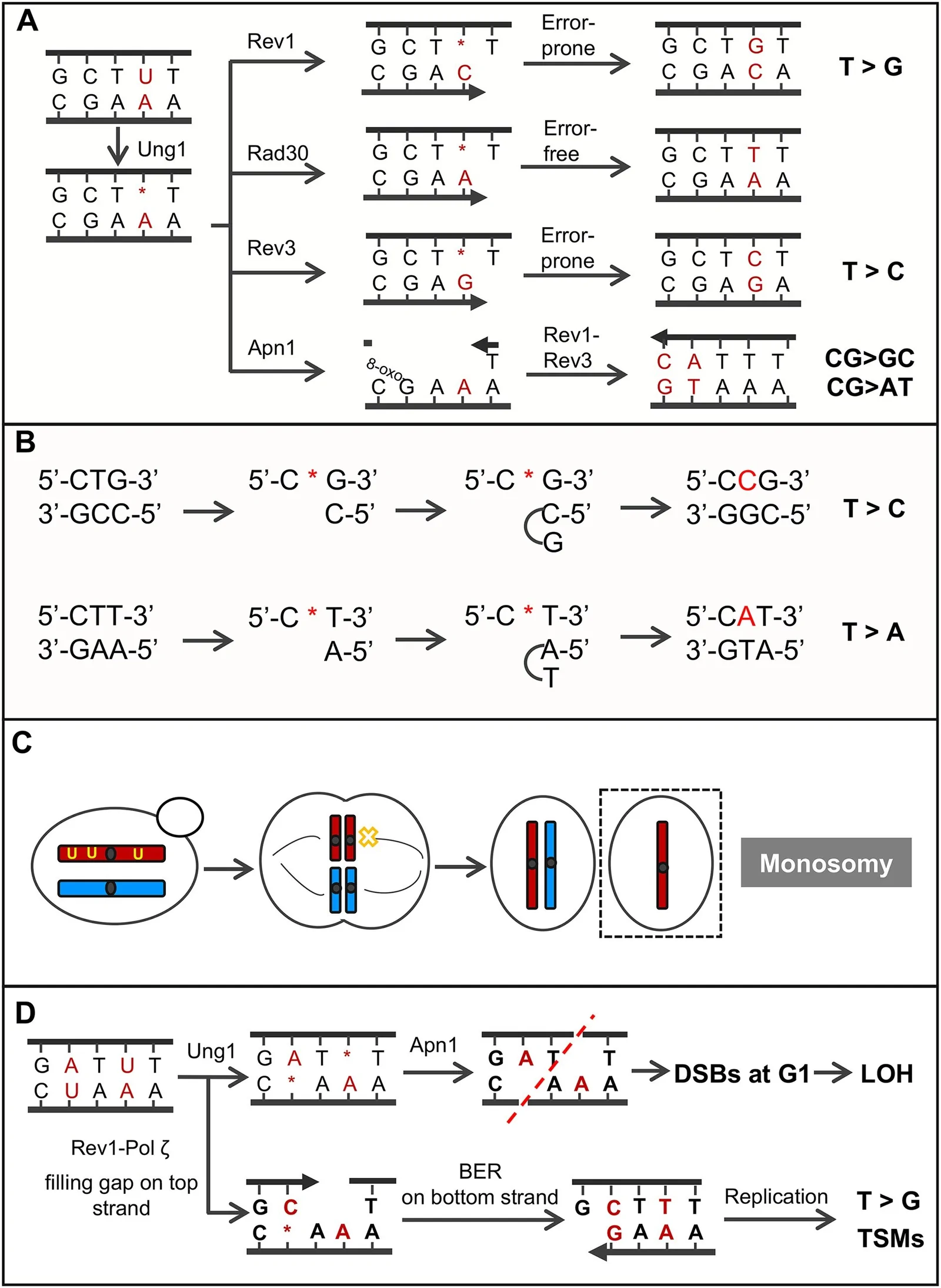
Proposed model for genomic alterations induced by reduced dUTPase expression. (**A**) Model for TLS-mediated mutation formation after uracil excision. Ung1 removes uracil and generates AP sites, which can be bypassed by TLS polymerases. Rev1 and Rev3 mediate error-prone bypass and contribute to T > G and T > C substitutions, respectively, whereas Rad30 promotes relatively error-free bypass. AP-site processing by Apn1 may also generate single-strand gaps. When these intermediates are associated with oxidative lesions such as 8-oxoG, Rev1 and Rev3 may participate in repair or bypass events that lead to CG > GC and CG > AT substitutions. (**B**) Model for sequence-context-dependent formation of T > C and T > A substitutions. Replication stalling at AP sites may reverse 3′ end of the nascent strand to generate primer-template misalignment. This gives rise to the enriched mutation contexts observed in Dut1-deficient cells. (**C**) Model for aneuploidy formation under reduced Dut1 expression. Uracil accumulation interferes with chromosome segregation, leading predominantly to chromosome loss. (**D**) Model for G1 phase DSBs and TSMs. Closely spaced AP sites or strand breaks on opposite strands may be converted into DSBs before DNA replication, thereby promoting G1-like LOH. In G1 phase, Rev1–Pol ζ-mediated gap filling on the upper strand, together with BER-mediated repair on the opposite strand, may generate TSMs, including T > G substitutions.

Second, the subsequent processing of AP sites triggers the formation of ssDNA gaps; TLS polymerases fill these transient gaps and inevitably introduce heterologous mutations at nonthymine positions (Fig. 7A). One evidence is CG-to-GC transversions dropped sharply in both *rev1*Δ and *rev3*Δ strains. This reflects the characteristic error-prone signature of the Rev1–Pol ζ complex, which is not restricted to a specific base lesion but stems from its critical role in extending mismatched termini [49]. *APN1* overexpression elevated the frequency of CG-to-GC transversions (Supplementary Fig. S6A), implying that the Rev1–Pol ζ complex actively generates these mutations during the downstream processing of AP sites.

Third, the massive influx of uracil-derived lesions may titrate the cellular pool of available Ung1 and Apn1, creating a severe repair bottleneck. This resource exhaustion leaves other heterologous spontaneous lesions—such as deaminated cytosines or oxidative 8-oxo-G—insufficiently serviced, leading to their fixation as C-to-T transitions and G-to-T transversions, respectively. The observation that *APN1* overexpression mitigated T-to-G mutation further validates the hypothesis that Apn1 is insufficient and represents a limiting bottleneck in *DUT1*-low cells.

Finally, given Rad30’s role in error-free bypass of 8-oxo-G [50], the G-to-T transversion surge following *RAD30* ablation demonstrates severe 8-oxo-G accumulation in *DUT1*-low cells. Because ssDNA lacks protective base stacking and hydrogen bonding, it is intrinsically hypersensitive to oxidation and deamination relative to dsDNA [51]. Consistently, the enriched C-to-T mutational signature in APOBEC3B-expressing strains supports a model wherein active AP site processing increases the exposure of vulnerable ssDNA, making it hyper-susceptible to secondary oxidative damage, predominantly in the form of 8-oxo-dG. Accordingly, the surge in G-to-T transversions upon *APN1* overexpression may be driven by 8-oxo-dG lesions accumulating on exposed ssDNA, which promote frequent adenine mispairing during replication bypass.

### TLS-dependent trade-off between SNVs and InDels

Analysis of the flanking sequences revealed distinct mutational signatures in *DUT1* low-expression strains: T-to-A substitutions were highly biased toward 5′-CTT-3′ motifs, while T-to-C transitions favored 5′-CTG-3′ and 5′-TTG-3′ contexts (Supplementary Fig. S2). We hypothesize that these biases arise from a primer-template misalignment mechanism during the bypass of Ung1-generated AP sites. Specifically, upon encountering an AP site, the replication fork stalls, potentially triggering the 3′ end of the nascent strand to transiently dissociate and loop back (reversion) (Fig. 7B). In this model, the nascent strand uses its own sequence as a local template for extension before realigning with the original template (Fig. 7B). Consequently, the base incorporated at the lesion site is complementary to the base immediately 3′ to the original T. This model is consistent with our data: in the 5′-CTT-3′ motif, the 3′ T dictates the incorporation of an A (T-to-A), whereas in 5′-CTG-3′, the 3′ G guides the incorporation of a C (T-to-C) (Fig. 7B). In contrast, T-to-G transversions—driven by the coordinated action of Rev1—bypass the AP site through direct dCMP incorporation, bypassing the need for nascent strand realignment (Fig. 7B). The observed 5′ sequence bias likely reflects the influence of upstream context on the efficiency of extension following these distinct TLS pathways.

Interestingly, we observed a significant elevation of InDels in *DUT1* low-expression strains, with the magnitude of increase exceeding that of SNVs. Similar to the trends observed for SNVs, Ung1 and Apn1 exerted promoting and inhibitory effects on InDels formation, respectively (Supplementary Fig. S7). Given that AP sites are known to facilitate DNA slippage [52], our findings suggest that replication slippage at Ung1-generated AP sites is a crucial driver of InDels in low Dut1 cells. Notably, the Rev1–Pol ζ complex appears to play a divergent role here compared to its effect on SNVs. We propose that the Rev1–Pol ζ complex bypasses AP sites by incorporating bases and extending the primer terminus (Fig. 7A); although this process is inherently error-prone and leads to base substitutions, it effectively prevents replication fork stalling, thereby mitigating the risk of catastrophic DNA slippage.

While the core principles of uracil-induced BER toxicity and replication stress are broadly conserved across eukaryotes, translating these yeast findings to mammalian systems warrants careful consideration of species-specific repair machinery. A key distinction lies in the complexity of TLS pathways. Mammalian cells harbor an expanded repertoire of specialized polymerases, including Pol ι, Pol κ, and PrimPol. These additional mammalian enzymes could offer enhanced functional redundancy or distinct misincorporation specificity when bypassing static AP sites or completing gap-filling synthesis across uracil-derived ssDNA regions. Consequently, a recognized limitation is that the precise mutational signatures and structural outcomes remain to be evaluated in human cells.

### Altered patterns of DNA recombination resulted from uracil overload

A defining hallmark of *DUT1*-low strains is a dramatic elevation in mitotic recombination (Fig. 2E). The drastic reduction in LOH (90%) and chromosomal rearrangement (86%) frequencies upon *UNG1* deletion provides definitive evidence that uracil–DNA glycosylase-mediated excision is the indispensable initiating step for these recombinogenic lesions. Moreover, our sector analysis revealed that ∼42% of crossovers originate from DSBs initiated during the S/G2 phases—a significant departure from the G1-dominance (70%) typically observed in spontaneous recombination [31]. This shift, coupled with S-phase delays (Fig. 1D) and ssDNA exposure (Fig. 6C), suggests that AP sites or their derivatives form formidable physical barriers to replication forks. However, while *APN1* deletion sharply exacerbates overall DSBs (Supplementary Fig. S3) and LOH frequencies (Fig. 4E), the recombination landscape in *apn1*Δ cells shifts predominantly toward I-LOH, contrasting with the T-LOH dominance characteristic of *DUT1*-low cells. This phenotypic divergence implies that DSBs induced directly by persistent, static AP sites are preferentially repaired via SDSA pathway. Conversely, DSBs arising from secondary lesions generated during downstream Apn1 processing are primarily resolved through dHR or BIR pathways—a model substantiated by the increased T-LOH proportion upon *APN1* overexpression (Supplementary Table S3). Thus, post-processing secondary lesions, rather than static AP sites *per se*, drive mitotic recombination under dUTPase deficiency. Aligning with the *E. coli* precedent where Ung-mediated excision causes Okazaki fragment-like fragmentation, we propose these secondary lesions manifest as extensive single-strand gaps from the clearance of clustered AP sites. These persistent gaps impede replication more severely than intact AP sites, mirroring the replication stress and T-LOH shifts induced by replicative DNA polymerase depletion in our prior work [17, 37].

Genome-wide mapping further reveals that recombination breakpoints are not stochastically distributed but are enriched at difficult-to-replicate regions, including Rrm3-binding sites and centromeres (Supplementary Table S5). Given that the Rrm3 helicase facilitates fork progression by displacing stable protein-DNA complexes [53, 54], this enrichment suggests that the specialized chromatin architecture or late-replicating nature of these loci renders them hypersensitive to uracil-induced stress. Parallel to this localized fragility, we observed dramatic fluctuations in rDNA and *CUP1* repeat copy numbers (Fig. 3D), with a decisive trend toward contraction. This recurrent contraction under chronic dUTPase limitation suggests a novel adaptive genomic plasticity: by streamlining repetitive arrays—inherent hotspots for replication-transcription conflicts—the cell minimizes the target size for uracil misincorporation and subsequent BER-mediated damage. This optimization reduces the cumulative probability of lethal fork collapse, presenting a clear selective advantage to preserve core genomic integrity.

### Synergistic contribution of dNTP pool imbalance to replication stress

Beyond the physical DNA lesions generated by uracil processing, our metabolomic profiling highlights a metabolic tier of toxicity: the systemic collapse of dNTP pool homeostasis (Supplementary Fig. S1). The >100-fold surge in intracellular dUTP, coupled with the marked depletion of downstream pyrimidine precursors (dUMP and dTMP) as well as dCTP and dGTP pools, imposes a severe metabolic bottleneck during DNA synthesis (Supplementary Fig. S1). This broad dNTP pool dysregulation may exacerbate replication stress through multiple synergistic mechanisms. First, the elevated dUTP/dTTP ratio allows dUTP to outcompete dTTP for binding at the active sites of replicative polymerases. Second, precursor starvation slows replication fork progression, directly contributing to the pronounced S-phase delay observed in low-*DUT1* cells (Fig. 1D). Third, imbalanced dNTP pools are well established to compromise the 3′-to-5′ exonuclease proofreading fidelity of replicative polymerases, promoting secondary base misincorporation across undamaged template DNA. Consequently, uracil-induced genome instability represents a compound phenotype driven jointly by physical BER-derived DNA lesions and systemic dNTP precursor starvation.

### Uracil overload jeopardizes chromosome disjunction, which frequently manifests as a high incidence of monosomy

Chromosomal aberrations, specifically monosomy, constitute the most significantly enriched class of genomic alterations in dUTPase-deficient cells. Crucially, the onset of aneuploidy in these cells follows a mechanistic trajectory distinct from that of DNA point mutations and recombination. While the latter are typically driven by Ung1-mediated uracil excision, chromosomal instability occurs independently of BER pathway.

We propose several nonexclusive mechanisms to account for this phenomenon (Fig. 7C): (i) centromeric chromatin disruption: extensive T-to-U substitutions may alter the epigenetic landscape of the centromere, potentially hindering the recruitment or stability of essential histone variants; (ii) kinetochore-microtubule instability: uracil accumulation within centromeric or pericentromeric DNA may compromise the structural integrity of kinetochore attachments, weakening interactions with spindle fibers and triggering unequal chromosome partitioning; (iii) cohesin dysregulation: the presence of uracil may interfere with the loading or maintenance of the cohesin complex, leading to premature sister chromatid separation or nondisjunction during mitosis.

Beyond driving sequence mutations and structural rearrangements, our conditional *DUT1*-limited yeast model serves as a robust platform for investigating the broader epigenetic and transcriptional consequences of genomic uracil incorporation. Noncanonical base accumulation can directly alter local chromatin conformation and DNA-protein binding affinities. For instance, the observed contraction of high-transcription repetitive loci, such as the rDNA and *CUP1* arrays, underscores how uracil-induced replication stress forces adaptive chromatin and copy-number remodeling. Future studies leveraging this system for transcriptomic profiling and chromatin accessibility mapping will further illuminate how nucleotide pool dysregulation intersects with epigenetic inheritance and gene regulation.

### dUTPdysgenesis drives catastrophic genomic instability and hybrid incompatibility

This work characterizes a unique phenomenon termed dUTPdysgenesis, in which the wild-type parent provides active Ung1 protein that attacks the uracil-preloaded genome of the other parent immediately following mating (Fig. 6D). Unlike MA isolates, dUTPdysgenesis is characterized by a high ratio of G1-phase recombinational lesions—with 78% of LOH events exhibiting 4:0 or complex gene conversion patterns (Supplementary Dataset S8)—and a high frequency of TSMs (Supplementary Dataset S9). This distinctive signature confirms that a rapid, cross-parental enzymatic assault prior to the first round of DNA replication drives catastrophic genomic instability.

We propose a model wherein the fusion of a uracil-preloaded haploid cell with a wild-type partner subjects the uracil-saturated genome to an immediate enzymatic shock via the influx of active Ung1. This rapid excision of uracil generates high-density, clustered AP sites and SSBs (Fig. 7D). In the G1 phase, the close proximity of SSBs may trigger the formation of DSBs, which subsequently manifest as lesions on both sister chromatids upon entry into S-phase. Furthermore, the processing of G1-phase AP sites likely generates extensive ssDNA gaps; the subsequent filling of these gaps by the error-prone Rev1–Pol ζ complex facilitates the fixation of mutations across both DNA strands before the cell initiates replication (Fig. 7D). This model is strongly supported by the observation that 55% of TSMs are T-to-G substitutions due to the C-rule activity of Rev1. This mutational profile differs fundamentally from that of the MA isolates, which exhibit a high frequency of nonuracil-site mutations, thereby highlighting the divergent genetic consequences of chronic uracil accumulation versus transient, high-intensity repair shocks. Notably, the frequent occurrence of TSMs in ultraviolet-irradiated and H_2_O_2_-treated G1 cells [55, 56] further underscores a universal paradigm for TSMs generation induced by both endogenous and exogenous genotoxic stressors.

Traditionally, hybrid incompatibility is explained by the Bateson–Dobzhansky–Muller model, where negative epistatic interactions between divergent alleles from two parent species result in reduced fitness [57, 58]. Most identified BDM genes involve protein-protein or protein-DNA mismatches, such as the HMR and LHR genes in *Drosophila* [59]. dUTPdysgenesis, however, represents a distinct class of “metabolic-enzymatic” incompatibility. Here, the incompatible factor is not a malfunctioning protein complex, but a metabolic state (uracil-preloaded DNA) that is inherently toxic when exposed to a universal repair enzyme (Ung1) from a mating partner. The catastrophic nature of dUTPdysgenesis is rooted in its timing. While S-phase lesions can often be bypassed by TLS polymerases or resolved via sister chromatid-mediated homologous recombination, G1-phase breaks—generated before a sister chromatid exists—force the cell to rely on the homologous chromosome from the other parent. This leads to the unidirectional loss of the uracil-rich genome. In nature, fluctuations in dUTPase activity—perhaps driven by environmental stress or nutrient limitation—could lead to transient uracil accumulation in certain populations. If these populations then attempt to hybridize with metabolic-normal individuals, dUTPdysgenesis could drive rapid genomic decay in the offspring, thereby facilitating reproductive isolation and potentially accelerating speciation. This mechanism highlights how ancestral metabolic history can be encoded directly into the chemical structure of the genome, dictating the success or failure of future genetic lineages.

In summary, our study establishes that Ung1-mediated BER is the primary driver of global genomic instability in dUTPase-deficient cells, effectively hijacking a normally protective pathway to generate intense replication stress and mutagenesis. This instability follows a bifurcated logic: an Ung1-dependent route facilitates DNA mutations and recombination, while a distinct, Ung1-independent mechanism triggers large-scale chromosomal aberrations. This metabolic-enzymatic crisis culminates in dUTPdysgenesis, a novel phenomenon where cross-parental Ung1 assault on uracil-preloaded genomes during the G1 phase induces catastrophic zygotic fragmentation and lethal genomic collapse. By resolving the dual-role paradox of uracil repair, these findings identify cellular Ung1 levels as a critical predictive biomarker for dUTP-disrupting chemotherapies such as 5-FU. Ultimately, we propose that exacerbating this aberrant repair process—rather than inhibiting it—offers a potent therapeutic strategy to maximize genomic catastrophe and enhance the efficacy of anti-cancer agents.

## Supplementary Material

gkag1072_Supplemental_Files

## Data Availability

The raw data of whole genome sequencing of yeast isoaltes were deposited in SRA database with the accession number PRJNA1461412.

## References

[1] Lindahl T . Instability and decay of the primary structure of DNA. Nature. 1993;362:709–15. 10.1038/362709a0 8469282

[2] Galperin MY, Moroz OV, Wilson KS et al. House cleaning, a part of good housekeeping. Mol Microbiol. 2006;59:5–19. 10.1111/j.1365-2958.2005.04950.x 16359314

[3] Blount BC, Mack MM, Wehr CM et al. Folate deficiency causes uracil misincorporation into human DNA and chromosome breakage: implications for cancer and neuronal damage. Proc Natl Acad Sci USA. 1997;94:3290–5. 10.1073/pnas.94.7.3290 PMC20362 9096386

[4] Galashevskaya A, Sarno A, Vågbø CB et al. A robust, sensitive assay for genomic uracil determination by LC/MS/MS reveals lower levels than previously reported. DNA Repair. 2013;12:699–706. 10.1016/j.dnarep.2013.05.002 23742752

[5] An Q, Robins P, Lindahl T et al. 5-Fluorouracil incorporated into DNA is excised by the Smug1 DNA glycosylase to reduce drug cytotoxicity. Cancer Res. 2007;67:940–5. 10.1158/0008-5472.CAN-06-2960 17283124

[6] Krokan HE, Bjørås M. Base excision repair. Cold Spring Harb Perspect Biol. 2013;5:a012583. 10.1101/cshperspect.a012583 PMC3683898 23545420

[7] Boiteux S, Guillet M. Abasic sites in DNA: repair and biological consequences in *Saccharomyces cerevisiae*. DNA Repair. 2004;3:1–12. 10.1016/j.dnarep.2003.10.002 14697754

[8] Saxena S, Nabel CS, Seay TW et al. Unprocessed genomic uracil as a source of DNA replication stress in cancer cells. Mol Cell. 2024;84:2036–2052.e7. 10.1016/j.molcel.2024.04.004 PMC11162326 38688279

[9] Warner HR, Duncan BK. *In vivo* synthesis and properties of uracil-containing DNA. Nature. 1978;272:32–4. 10.1038/272032a0 342978

[10] Tye B-K, Nyman P-O, Lehman I et al. Transient accumulation of Okazaki fragments as a result of uracil incorporation into nascent DNA. Proc Natl Acad Sci USA. 1977;74:154–7. 10.1073/pnas.74.1.154 PMC393216 319455

[11] Mortusewicz O, Haslam J, Gad H et al. Uracil-induced replication stress drives mutations, genome instability, anti-cancer treatment efficacy, and resistance. Mol Cell. 2025;85:1897–906. 10.1016/j.molcel.2025.04.015 40378828

[12] Dos Santos R S, Daures M, Philippi A et al. dUTPase (DUT) is mutated in a novel monogenic syndrome with diabetes and bone marrow failure. Diabetes. 2017;66:1086–96. 10.2337/db16-0839 28073829

[13] Owiti N, Wei S, Bhagwat AS et al. Unscheduled DNA synthesis leads to elevated uracil residues at highly transcribed genomic loci in *Saccharomyces cerevisiae*. PLoS Genet. 2018;14:e1007516. 10.1371/journal.pgen.1007516 PMC6063437 30016327

[14] Guillet M, Van D, Kemp PA et al. dUTPase activity is critical to maintain genetic stability in *Saccharomyces cerevisiae*. Nucleic Acids Res. 2006;34:2056–66. 10.1093/nar/gkl139 PMC1440884 16617146

[15] Zhang K, Sui Y, Li WL et al. Global genomic instability caused by reduced expression of DNA polymerase ε in yeast. Proc Natl Acad Sci USA. 2022;119:e2119588119. 10.1073/pnas.2119588119 PMC8944251 35290114

[16] Zheng D-Q, Wang P-M, Chen J et al. Genome sequencing and genetic breeding of a bioethanol *Saccharomyces cerevisiae* strain YJS329. BMC Genomics. 2012;13:479. 10.1186/1471-2164-13-479 PMC3484046 22978491

[17] Zheng D-Q, Zhang K, Wu X-C et al. Global analysis of genomic instability caused by DNA replication stress in *Saccharomyces cerevisiae*. Proc Natl Acad Sci USA. 2016;113:E8114–21. 10.1073/pnas.1618129113 PMC5167146 27911848

[18] Gueldener U, Heinisch J, Koehler G et al. A second set of loxP marker cassettes for Cre-mediated multiple gene knockouts in budding yeast. Nucleic Acids Res. 2002;30:23e. 10.1093/nar/30.6.e23 PMC101367 11884642

[19] Gietz RD, Schiestl RH. High-efficiency yeast transformation using the LiAc/SS carrier DNA/PEG method. Nat Protoc. 2007;2:31–4. 10.1038/nprot.2007.13 17401334

[20] Sui Y, Qi L, Zhang K et al. Analysis of APOBEC-induced mutations in yeast strains with low levels of replicative DNA polymerases. Proc Natl Acad Sci USA. 2020;117:9440–50. 10.1073/pnas.1922472117 PMC7196835 32277034

[21] Zhu Y, Tong X, Xue J et al. Phospholipid biosynthesis modulates nucleotide metabolism and reductive capacity. Nat Chem Biol. 2025;21:35–46. 10.1038/s41589-024-01689-z 39060393

[22] Sui Y, Qi L, Wu J-K et al. Genome-wide mapping of spontaneous genetic alterations in diploid yeast cells. Proc Natl Acad Sci USA. 2020;117:28191–200. 10.1073/pnas.2018633117 PMC7668089 33106417

[23] Bolger AM, Lohse M, Usadel B. Trimmomatic: a flexible trimmer for Illumina sequence data. Bioinformatics. 2014;30:2114–20. 10.1093/bioinformatics/btu170 PMC4103590 24695404

[24] Li H . Aligning sequence reads, clone sequences and assembly contigs with BWA-MEM. 2013, arXiv. preprint arXiv:1303.3997, 9 May 2022, preprint: not peer reviewed.

[25] Li H, Handsaker B, Wysoker A et al. The sequence alignment/map format and SAMtools. Bioinformatics. 2009;25:2078–9. 10.1093/bioinformatics/btp352 PMC2723002 19505943

[26] Koboldt DC, Zhang Q, Larson DE et al. VarScan 2: somatic mutation and copy number alteration discovery in cancer by exome sequencing. Genome Res. 2012;22:568–76. 10.1101/gr.129684.111 PMC3290792 22300766

[27] Qi L, Li K-J, Tang X-X et al. Loss of Pol32, a subunit of DNA polymerases δ and ζ, leads to different patterns of genome stability than direct impairment of these individual polymerases. mBio. 2026;:e00531–26. 10.1128/mbio.00531-26 PMC13170350 41995324

[28] McGinty RJ, Rubinstein RG, Neil AJ et al. Nanopore sequencing of complex genomic rearrangements in yeast reveals mechanisms of repeat-mediated double-strand break repair. Genome Res. 2017;27:2072–82. 10.1101/gr.228148.117 PMC5741057 29113982

[29] Sedlazeck FJ, Rescheneder P, Smolka M et al. Accurate detection of complex structural variations using single-molecule sequencing. Nat Methods. 2018;15:461–8. 10.1038/s41592-018-0001-7 PMC5990442 29713083

[30] Li H, Handsaker B, Wysoker A et al. The Sequence Alignment/Map format and SAMtools. Bioinformatics. 2009;25:2078–9. 10.1093/bioinformatics/btp352 PMC2723002 19505943

[31] St. Charles J, Petes TD. High-resolution mapping of spontaneous mitotic recombination hotspots on the 1.1 Mb arm of yeast chromosome IV. PLoS Genet. 2013;9:e1003434. 10.1371/journal.pgen.1003434 PMC3616911 23593029

[32] Zheng DQ, Petes TD. Genome instability induced by low levels of replicative DNA polymerases in yeast. Genes. 2018;9:539. 10.3390/genes9110539 PMC6267110 30405078

[33] Qi L, Sui Y, Tang X-X et al. Shuffling the yeast genome using CRISPR/Cas9-generated DSBs that target the transposable Ty1 elements. PLoS Genet. 2023;19:e1010590. 10.1371/journal.pgen.1010590 PMC9879454 36701275

[34] Sampaio NM, Ajith V, Watson RA et al. Characterization of systemic genomic instability in budding yeast. Proc Natl Acad Sci USA. 2020;117:28221–31. 10.1073/pnas.2010303117 PMC7668020 33106418

[35] Vijayan N, Joshi S, Sarath P et al. Loss of Heterozygosity associated with ubiquitous environments in yeast. PLoS Genet. 2025;21:e1011692. 10.1371/journal.pgen.1011692 PMC12068580 40354302

[36] Jinks-Robertson S, Petes TD. Mitotic recombination in yeast: what we know and what we don’t know. Curr Opin Genet Dev. 2021;71:78–85. 10.1016/j.gde.2021.07.002 PMC8671248 34311384

[37] Zhang K, Sui Y, Li W-L et al. Global genomic instability caused by reduced expression of DNA polymerase ε in yeast. Proc Natl Acad Sci USA. 2022;119:e2119588119. 10.1073/pnas.2119588119 PMC8944251 35290114

[38] Symington LS, Rothstein R, Lisby M. Mechanisms and regulation of mitotic recombination in *Saccharomyces cerevisiae*. Genetics. 2014;198:795–835. 10.1534/genetics.114.166140 PMC4224172 25381364

[39] Meng X, Wei L, Peng XP et al. Sumoylation of the DNA polymerase ε by the Smc5/6 complex contributes to DNA replication. PLoS Genet. 2019;15:e1008426. 10.1371/journal.pgen.1008426 PMC6876774 31765372

[40] Yan C, Wang Y-K, Xiong Y-R et al. The roles of NHEJ and TLS pathways in genomic alterations and phenotypic evolution in the yeast *Yarrowia lipolytica*. Appl Microbiol Biotechnol. 2025;109:183. 10.1007/s00253-025-13575-2 PMC12354579 40813729

[41] Zheng D-Q, Wang Y-T, Zhu Y-X et al. Uncovering bleomycin-induced genomic alterations and underlying mechanisms in the yeast *Saccharomyces cerevisiae*. Appl Environ Microb. 2022;88:e01703–21. 10.1128/AEM.01703-21 PMC8788679 34731050

[42] Kiktev DA, Sheng Z, Lobachev KS et al. GC content elevates mutation and recombination rates in the yeast *Saccharomyces cerevisiae*. Proc Natl Acad Sci USA. 2018;115:E7109–18. 10.1073/pnas.1807334115 PMC6064992 29987035

[43] Nelson JR, Lawrence CW, Hinkle DC. Deoxycytidyl transferase activity of yeast REV1 protein. Nature. 1996;382:729–31. 10.1038/382729a0 8751446

[44] Johnson RE, Prakash S, Prakash L. Efficient bypass of a thymine-thymine dimer by yeast DNA polymerase, Polη. Science. 1999;283:1001–4. 10.1126/science.283.5404.1001 9974380

[45] Martin SK, Wood RD. DNA polymerase ζ in DNA replication and repair. Nucleic Acids Res. 2019;47:8348–61. 10.1093/nar/gkz705 PMC6895278 31410467

[46] Nikkila J, Kumar R, Campbell J et al. Elevated APOBEC3B expression drives a kataegic-like mutation signature and replication stress-related therapeutic vulnerabilities in p53-defective cells. Br J Cancer. 2017;117:113–23. 10.1038/bjc.2017.133 PMC5520199 28535155

[47] Sui Y, Qi L, Zhang K et al. Analysis of APOBEC-induced mutations in yeast strains with low levels of replicative DNA polymerases. Proc Natl Acad Sci USA. 2020;117:9440–50. 10.1073/pnas.1922472117 PMC7196835 32277034

[48] Sui Y, Epstein A, Dominska M et al. Ribodysgenesis: sudden genome instability in the yeast *Saccharomyces cerevisiae* arising from RNase H2 cleavage at genomic-embedded ribonucleotides. Nucleic Acids Res. 2022;50:6890–902. 10.1093/nar/gkac536 PMC9262587 35748861

[49] Northam MR, Robinson HA, Kochenova OV et al. Participation of DNA polymerase ζ in replication of undamaged DNA in *Saccharomyces cerevisiae*. Genetics. 2010;184:27–42. 10.1534/genetics.109.107482 PMC2815923 19841096

[50] Haracska L, Yu S-L, Johnson RE et al. Efficient and accurate replication in the presence of 7,8-dihydro-8-oxoguanine by DNA polymerase η. Nat Genet. 2000;25:458–61. 10.1038/78169 10932195

[51] Roberts SA, Sterling J, Thompson C et al. Clustered mutations in yeast and in human cancers can arise from damaged long single-strand DNA regions. Mol Cell. 2012;46:424–35. 10.1016/j.molcel.2012.03.030 PMC3361558 22607975

[52] Kim N, Jinks-Robertson S. dUTP incorporation into genomic DNA is linked to transcription in yeast. Nature. 2009;459:1150–3. 10.1038/nature08033 PMC2730915 19448611

[53] Ivessa AS, Lenzmeier BA, Bessler JB et al. The *Saccharomyces cerevisiae* helicase Rrm3p facilitates replication past nonhistone protein-DNA complexes. Mol Cell. 2003;12:1525–36. 10.1016/S1097-2765(03)00456-8 14690605

[54] Muñoz-Galván S, Garcia-Rubio M, Ortega P et al. A new role for Rrm3 in repair of replication-born DNA breakage by sister chromatid recombination. PLoS Genet. 2017;13:e1006781. 10.1371/journal.pgen.1006781 PMC5438189 28475600

[55] Zhu Y-X, Li K-J, He M et al. Mitotic recombination events and single-base mutations induced by ultraviolet light in G1-arrested yeast cells. Proc Natl Acad Sci USA. 2025;122:e2518046122. 10.1073/pnas.2518046122 PMC12557804 41091767

[56] Qi L, Zhang K, Zheng DQ et al. Detection of “hidden” mitotic crossovers by long-read DNA sequencing. Proc Natl Acad Sci USA. 2026;123:e2615875123. 10.1073/pnas.2615875123 PMC13534622 42647135

[57] Li C, Wang Z, Zhang J. Toward genome-wide identification of Bateson–Dobzhansky–Muller incompatibilities in yeast: a simulation study. Genome Biol Evol. 2013;5:1261–72. 10.1093/gbe/evt091 PMC3730343 23742870

[58] Cutter AD . The polymorphic prelude to Bateson–Dobzhansky–Muller incompatibilities. Trends Ecol Evol. 2012;27:209–18. 10.1016/j.tree.2011.11.004 22154508

[59] Satyaki P, Cuykendall TN, Wei KH et al. The Hmr and Lhr hybrid incompatibility genes suppress a broad range of heterochromatic repeats. PLoS Genet. 2014;10:e1004240. 10.1371/journal.pgen.1004240 PMC3961192 24651406

