# Supplementary material for: Uracil overload triggers global genome instability and dUTPdysgenesis
Source: Nucleic Acids Res. 2026 Oct 27;54(20):gkag1072. doi: 10.1093/nar/gkag1072 (PMC13710866; doi:10.1093/nar/gkag1072)
Supplement: Supplementary file 1 [file gkag1072_supplemental_files.zip › Supplemental file.pdf]

Uracil overload triggers global genome instability and dUTPdysgenesis

Qiao-Ling Yin[1,#], Min He[1,#], Ke Zhang[2,#], Kejing Li[1], Yeke Wang[2], Wan-Jing Ding[1], Yibin Zhu[4], Cunqi Ye[3], Dao-Qiong Zheng[1,*]

[#] These authors contributed equally to this work.

[*] To whom correspondence should be addressed. Email: zhengdaoqiong@zju.edu.cn

[1] State Key Laboratory (SKL) of Biobased Transportation Fuel Technology, Ocean College, Zhejiang University, Zhoushan, 321006, China

[2] College of Life Science, Zhejiang University, Hangzhou, 310058, China

[3] Zhejiang Provincial Key Laboratory for Cancer Molecular Cell Biology, Life Sciences Institute, Zhejiang University, Hangzhou, China

[4] Institute of Medical Genetics and Development, Key Laboratory of Reproductive Genetics (Ministry of Education) and Department of Reproductive Endocrinology, Women's Hospital, School of Medicine, Zhejiang University, Hangzhou, China.

Supplemental text

Strain construction

Construction of yeast strains

To generate the *kanMX6-GAL1-3×HA-DUT1* allele at the native *DUT1* locus, the endogenous *DUT1* promoter was replaced with a *kanMX6-GAL1-3×HA* cassette by homologous recombination. Within the *DUT1* coding sequence, only the native ATG start codon was replaced. The downstream *DUT1* coding sequence, lacking its native ATG, was retained and fused in-frame to the upstream *3×HA* coding sequence through the designed junction. Translation initiates from the ATG placed upstream of the *3×HA* coding sequence in the *GAL1-3×HA* module, producing an N-terminally *3×HA*-tagged Dut1 protein.

Two synthetic plasmids, G-pqll-DUT1 and pKQLL (lab stock), were synthesized by Qingdao BGI and used as PCR templates for construction of the *kanMX6-GAL1-3×HA-DUT1* replacement cassette. G-pqll-DUT1 contains a synthetic DNA fragment derived from the *DUT1* locus, including the *DUT1* upstream homology region, the downstream *DUT1* coding sequence lacking the native ATG, and designed overlap sequences required for cassette assembly. This plasmid was used as the template to amplify *DUT1*-derived homologous fragments. pKQLL contains the *kanMX6-GAL1-3×HA* module and was used as the template to amplify the fragment carrying the *kanMX6* selectable marker, the *GAL1* promoter, and the *3×HA* coding sequence.

Three DNA fragments were prepared for overlap PCR assembly. Fragment 1 was amplified from G-pqll-DUT1 using primers kup250-ovlp-S and kup250-ovlp-A (Table S2). This fragment contained the *DUT1* upstream homology region and an overlap sequence required for fusion to the 5' side of the *kanMX6-GAL1-3×HA* module. Fragment 2 was amplified from G-pqll-DUT1 using primers kdn248-ovlp-S and kdn248-ovlp-A (Table S2). This fragment starts immediately downstream of the native *DUT1* ATG start codon and contains the retained downstream *DUT1* coding sequence required for in-frame fusion with the upstream *3×HA* coding sequence. Fragment 3 was amplified from pKQLL using primers dprom-ovlp-S and dprom-ovlp-A (Table S3). This fragment contained the *kanMX6-GAL1-3×HA* module. These

three purified PCR products were mixed and assembled by overlap PCR. In the first step, the fragments were joined through their designed complementary ends by 8 cycles of overlap extension PCR. In the second step, the assembled full-length product was amplified by 38 PCR cycles using the outer primers dprom-GPQLL-S and dprom-GPQLL-A. The resulting linear PCR product was used as the *kanMX6-GAL1-3×HA-DUT1* replacement cassette for yeast transformation. The transformants were selected on HG medium containing 300 mg/L G418. Correct replacement of the endogenous *DUT1* promoter and the native *DUT1* ATG start codon was verified by diagnostic PCR using primers flanking the target locus (Table S2).

The *kanMX6-GAL1-3×HA-DUT1* cassette was introduced into SY165, YJSH1, and SY166 to generate WDU, YJDU and YDU, respectively. WDU was then mated with YJDU or YDU on HG his$^-$ met$^-$ plates to generate the diploid strains DU and WYDU, respectively.

DNA repair deficiency mutants were constructed using SY165 and YJSHU as the haploid parental strains (Table S1). With the exception of the *RAD52*-deletion derivatives, these derivatives were generated by a two-step strategy. First, the indicated repair gene was deleted in the appropriate haploid parental strain. Second, the resulting deletion strain was transformed with the *kanMX6-GAL1-3×HA-DUT1* replacement cassette to generate a repair-gene-deletion strain with reduced *DUT1* expression. For the *RAD52*-deletion derivatives, the order was reversed: the parental strain was first transformed with the *kanMX6-GAL1-3×HA-DUT1* replacement cassette, followed by deletion of the *RAD52* coding sequence.

For DNA repair-gene deletion, *URA3*-based deletion cassettes were amplified from pUG72 using long primers carrying target-specific homology arms. These deletion cassettes were introduced into haploid strains carrying a *URA3* auxotrophic marker, allowing selection on SD-U medium. For example, the *rev1::URA3* cassette was amplified from pUG72 using long primers containing *REV1* upstream and downstream homology arms (Table S2). This cassette was introduced into a ura3 mutant haploid strain to replace the *REV1* coding sequence with *URA3* by homologous recombination. Correct insertion at the *REV1* locus and deletion of the

REV1 coding sequence were verified by diagnostic PCR. The same *URA3*-based strategy was used to delete other repair genes, including *REV3*, *RAD30*, *UNG1*, *APN1*, *CHL1*, *CTF18*, *RAD51*, and *RAD52*, using gene-specific homology arms. The resulting haploid strains carrying both the repair-gene deletion and the *kanMX6-GAL1-3×HA-DUT1* allele were mated with the corresponding opposite mating-type strains on HG his− met− plates to generate diploid derivative strains.

For construction of DUU-APN1, the pSH86-APN1 plasmid (lab stock) carrying *APN1* under the *TEF1* promoter and a *URA3* selection marker was transformed into DUU cells. Transformants were selected on HG U- plates and maintained under uracil-dropout selection. DUU-APN1 mutation-accumulation isolates were propagated on LG U− plates. LG U- medium contained 0.67% yeast nitrogen base, 3% raffinose, 0.005% galactose and 0.14% SD supplement lacking uracil; HG U- medium had the same composition except with 0.1% galactose.

Table S1. Yeast strains used in this study.

Strain	Background	Genotype	Construction
SY165	W303-1A	*MAT*a *leu2-3,112 his3-11,15 ura3-1 ade2-1 trp1-1 can1-100 rad5 spo11::loxp*	Lab stock
SY166	YJM789	*MATα ade2-1 ura3 gal2 ho::hisG IV1510386::SUP4-o spo11::loxp*	Lab stock
YJSH1	YJS329	*MATα met17 spo11*	Lab stock
YJSHU	YJS329	*MATα ura3 met17 spo11*	Lab stock
WSD	W303-1A	*MAT*a *leu2-3,112 his3-11,15 ura3-1 ade2-1 trp1-1 can1-100 rad5 spo11::loxp 3×HA-DUT1*	*kanMX6-GAL1* removed from WDU by selection on galactose-free medium
WDU	W303-1A	*MAT*a *leu2-3,112 his3-11,15 ura3-1 ade2-1 trp1-1 can1-100 rad5 spo11::loxp kanMX6-GAL1-3×HA-DUT1*	Replaced the promoter of *DUT1* with p*GAL1* in SY165
YJSU	YJS329	*MATα met17 spo11 kanMX6-GAL1-3×HA-DUT1*	Replaced the promoter of *DUT1* with p*GAL1* in YJSU
YJSUU	YJS329	*MATα ura3 met17 spo11 kanMX6-GAL1-3×HA-DUT1*	Replaced the promoter of *DUT1* with p*GAL1* in YJSUU
WDUΔapn1	W303-1A	*MAT*a *leu2-3,112 his3-11,15 ura3-1 ade2-1 trp1-1 can1-100 rad5 spo11::loxp apn1Δ::URA3 kanMX6-GAL1-3×HA-DUT1*	*apn1Δ::URA3* deletion followed by p*GAL-DUT1* integration in SY165
WDUΔung1	W303-1A	*MAT*a *leu2-3,112 his3-11,15 ura3-1 ade2-1 trp1-1 can1-100 rad5 spo11::loxp ung1Δ::URA3 kanMX6-GAL1-3×HA-DUT1*	*ung1Δ::URA3* deletion followed by p*GAL-DUT1* integration in SY165
EG0	W303-1A× YJS329	*MAT*a*/MATα leu2-3,112/+ his3-11,15/+ ura3-1/+ ade2-1/+ trp1-1/+ can1-100/+ rad5/+ spo11::loxP/spo11 + /met17*	Mating of SY165 and YJSH1
DU	W303-1A× YJS329	*MAT*a*/MATα leu2-3,112/+ his3-11,15/+ ura3-1/+ ade2-1/+ trp1-1/+ can1-100/+ rad5/+ spo11::loxP/spo11 +/met17 kanMX6-GAL1-3×HA-DUT1/ kanMX6-GAL1-3×HA-DUT1*	Mating of SY165- and YJSU
DUU	W303-1A× YJS329	*MAT*a*/MATα leu2-3,112/+ his3-11,15/+ ura3-1/ura3 ade2-1/+ trp1-1/+ can1-100/+ rad5/+ spo11::loxP/spo11 + /met17 kanMX6-GAL1-3×HA-DUT1/ kanMX6-GAL1-3×HA-DUT1*	Mating of WDU and YJSUU
DUUΔung1	W303-1A× YJS329	*MAT*a*/MATα leu2-3,112/+ his3-11,15/+ ura3-1/ura3 ade2-1/+ trp1-1/+ can1-100/+ rad5/+ spo11::loxP/spo11 + /met17 ung1Δ::URA3/ ung1Δ::URA3 kanMX6-GAL1-3×HA-DUT1/ kanMX6-GAL1-3×HA-DUT1*	Mating of matched *ung1Δ::URA3* p*GAL-DUT1* haploids
DUUΔapn1	W303-1A× YJS329	*MAT*a*/MATα leu2-3,112/+ his3-11,15/+ ura3-1/ura3 ade2-1/+ trp1-1/+ can1-100/+ rad5/+ spo11::loxP/spo11 + /met17 apn1Δ::URA3/ apn1Δ::URA3 kanMX6-GAL1-3×HA-DUT1/ kanMX6-GAL1-3×HA-DUT1*	Mating of matched *apn1Δ::URA3* p*GAL-DUT1* haploids
DUUΔrev1	W303-1A× YJS329	*MAT*a*/MATα leu2-3,112/+ his3-11,15/+ ura3-1/ura3 ade2-1/+ trp1-1/+ can1-100/+ rad5/+ spo11::loxP/spo11 + /met17 rev1Δ::URA3/ rev1Δ::URA3 kanMX6-GAL1-3×HA-DUT1/ kanMX6-GAL1-3×HA-DUT1*	Mating of matched *rev1Δ::URA3* p*GAL-DUT1* haploids
DUUΔrev3	W303-1A× YJS329	*MAT*a*/MATα leu2-3,112/+ his3-11,15/+ ura3-1/ura3 ade2-1/+ trp1-1/+ can1-100/+ rad5/+ spo11::loxP/spo11 + /met17 rev3Δ::URA3/ rev3Δ::URA3 kanMX6-GAL1-3×HA-DUT1/ kanMX6-GAL1-3×HA-DUT1*	Mating of matched *rev3Δ::URA3* p*GAL-DUT1* haploids
DUUΔrad30	W303-1A× YJS329	*MAT*a*/MATα leu2-3,112/+ his3-11,15/+ ura3-1/ura3 ade2-1/+ trp1-1/+ can1-100/+ rad5/+ spo11::loxP/spo11 + /met17 rad30Δ::URA3/ rad30Δ::URA3 kanMX6-GAL1-3×HA-DUT1/*	Mating of matched *rad30Δ::URA3* p*GAL-DUT1* haploids

		kanMX6-GAL1-3×HA-DUT1	
DUUΔrad51	W303-1A× YJS329	*MAT*a*/MATα leu2-3,112/+ his3-11,15/+ ura3-1/ura3 ade2-1/+ trp1-1/+ can1-100/+ rad5/+ spo11::loxP/spo11 + /met17 rad51Δ::URA3/ rad51Δ::URA3 kanMX6-GAL1-3×HA-DUT1/ kanMX6-GAL1-3×HA-DUT1*	Mating of matched *rad51Δ::URA3* p*GAL-DUT1* haploids
DUUΔrad52	W303-1A× YJS329	*MAT*a*/MATα leu2-3,112/+ his3-11,15/+ ura3-1/ura3 ade2-1/+ trp1-1/+ can1-100/+ rad5/+ spo11::loxP/spo11 + /met17 rad52Δ::URA3/ rad52Δ::URA3 kanMX6-GAL1-3×HA-DUT1/ kanMX6-GAL1-3×HA-DUT1*	Mating of matched *rad52Δ::URA3* p*GAL-DUT1* haploids
DUUΔctf18	W303-1A× YJS329	*MAT*a*/MATα leu2-3,112/+ his3-11,15/+ ura3-1/ura3 ade2-1/+ trp1-1/+ can1-100/+ rad5/+ spo11::loxP/spo11 + /met17 ctf18Δ::URA3/ ctf18Δ::URA3 kanMX6-GAL1-3×HA-DUT1/ kanMX6-GAL1-3×HA-DUT1*	Mating of matched *ctf18Δ::URA3* p*GAL-DUT1* haploids
DUUΔchl1	W303-1A× YJS329	*MAT*a*/MATα leu2-3,112/+ his3-11,15/+ ura3-1/ura3 ade2-1/+ trp1-1/+ can1-100/+ rad5/+ spo11::loxP/spo11 + /met17 chl1Δ::URA3/chl1Δ::URA3 kanMX6-GAL1-3×HA-DUT1/ kanMX6-GAL1-3×HA-DUT1*	Mating of matched *chl1Δ::URA3* p*GAL-DUT1* haploids
DUU-APN1	W303-1A× YJS329	*MAT*a*/MATα leu2-3,112/+ his3-11,15/+ ura3-1/ura3 ade2-1/+ trp1-1/+ can1-100/+ rad5/+ spo11::loxP/spo11 + /met17 kanMX6-GAL1-3×HA-DUT1/ kanMX6-GAL1-3×HA-DUT1* [pSH86-APN1(*APN1,URA3)*]	DUU transformed with pSH86-APN1
YXM61	W303-1A	*MAT*a *leu2-3,112 his3-11,15 ura3-1 ade2-1 trp1-1 can1-100 rad5-535 RAD52-YFP*	Meng XZ Lab
YXM61D	W303-1A	*MAT*a *leu2-3,112 his3-11,15 ura3-1 ade2-1 trp1-1 can1-100 rad5-535 RAD52-YFP kanMX6-GAL1-3×HA-DUT1*	p*GAL-DUT1* integrated at the *DUT1* locus in YXM61
YDU	YJM789	*MATα ade2- ura3 spo11::loxP IV1510386::SUP4-o kanMX6-GAL1-3×HA-DUT1*	p*GAL-DUT1* integrated at the *DUT1* locus in SY166
WYDU	W303-1A× YJM789	*MAT*a*/MATα ade2-1/ade2-1 ura3-1/ura3 spo11::loxP/spo11::loxP leu2-3,112/+ his3-11,15/+ trp1-1/+ can1-100/+ rad5/+ gal2/+ ho::hisG/+ IV1510386::SUP4-o/+ kanMX6-GAL1-3×HA-DUT1/ kanMX6-GAL1-3×HA-DUT1/ kanMX6-GAL1-3×HA-DUT1*	Mating of WDU and YDU
WYUU	W303-1A× YJM789	*MAT*a*/MATα ade2-1/ade2-1 ura3-1/ura3 spo11::loxP/spo11::loxP leu2-3,112/+ his3-11,15/+ trp1-1/+ can1-100/+ rad5/+ gal2/+ ho::hisG/+ IV1510386::SUP4-o/+ ung1Δ::URA3/+ kanMX6-GAL1-3×HA-DUT1/+*	Mating of WDUΔung1 and SY166
EG0-A3B	W303-1A× YJS329	*MAT*a*/MATα leu2-3,112/+ his3-11,15/+ ura3-1/+ ade2-1/+ trp1-1/+ can1-100/+ rad5/+ spo11::loxP/spo11 + /met17* [pSR440, (*APOBEC3B*, Hyg^R)]	EG0 transformed with pSR440
DU-A3B	W303-1A× YJS329	*MAT*a*/MATα leu2-3,112/+ his3-11,15/+ ura3-1/+ ade2-1/+ trp1-1/+ can1-100/+ rad5/+ spo11::loxP/spo11 +/met17 kanMX6-GAL1-3×HA-DUT1/ kanMX6-GAL1-3×HA-DUT1* [pSR440, (*APOBEC3B*, Hyg^R)]	DU transformed with pSR440

p*GAL-DUT1* denotes the *kanMX6-GAL1-3×HA-DUT1* cassette.

Table S2. Primers used in this study

Primer	Sequences	Purpose
dprom-GPQLL-S	TTGTCAAGCTAAGTTCAG	For construction of the *kanMX6-GAL1-3×HA-DUT1* cassette
dprom-GPQLL-A	TGCCAATGTTCAGTCTAC	
kup250-ovlp-S	AGGTCGCCTGACGCATATACAGGTGAGCCA GTGAGTTG	
kup250-ovlp-A	ATCATGCGTCAATCGTATGTGAATGCTGGTC GCTATACTGCTGTCGATTCGATACTAACG	
kdn248-ovlp-S	ACTATGCGGGCTATCCGTATGACGTCCCGGA CTATGCAGGATCCTATCCATATGACGTTC	
kdn248-ovlp-A	TATCACGAGGCGAGCTCGGAGTCAGTGAGC GAGGAA	
dprom-ovlp-S	CAGTATAGCGACCAGCAT	
dprom-ovlp-A	CCTGCATAGTCCGGGACG	
OT-DUT1-S	AAGACATTGGATGAGGTTGCC	To verify integration of the *kanMX6-GAL1-3×HA-DUT1* cassette
v-kMx(0912EG)-A	CGCAGACCGATACCAGGAT	
OT-DUT1-A	AGTTAGTTACCAGTGCTACCAA	
MAT-F	AGTCACATCAAGATCGTTTATGG	To verify successful mating
MAT-a	ACTCCACTTCAAGTAAGAGTTTG	
MAT-α	GCACGGAATATGGGACTACTTCG	
o1-rev1-S	TTTCTCAAAATAAATCGATACTGCATTTCTAG GCATATCCAGCTGCAGGTCGACAACCC	For construction of the *rev1*::*URA3* cassette by amplifying the *URA3* fragment from plasmid pUG72 with long primers carrying homologous arms
o2-rev1-A	TTATTTTTTTACTTAATTTTATTTAATTGTCC ATTGTGTCAGTGGATCTGATATCACC	
v-PUG72-A	GACGAAGTTGTAAGTGGTGGAT	For internal verification of the *URA3* selectable marker
OT-rev1-S	CTACGAACTTGCTGATGTCCTT	To verify integration of the *rev1*::*URA3* cassette
In-rev1-S	TTGGCGGTGGCGAAGATT	
In-rev1-A	TACTGGTGACTGCTGTTCTACT	
o1-rev3-S	CAAGAGAAAGTATTTGAGTCAATACAAAAC TACAAGTTGTGGCTGCAGGTCGACAACCC	For construction of the *rev3*::*URA3* cassette by amplifying the *URA3* fragment from plasmid pUG72 with long primers carrying homologous arms
o2-rev3-A	ACAAATAACTACTCATCATTTTGCGAGACAT ATCTGTGTCTAGTGGATCTGATATCACC	
OT-rev3-S	AAGAATCCCTGTGGTCTCCTAC	To verify integration of the *rev3*::*URA3* cassette
In-rev3-S	ACGACACAATACAGAGCGATAC	
In-rev3-A	CAACAGAGACATCAGCGACAA	
OT2-ung1-S	GCAGTGAGGTCGTTGAAGATT	For construction of the *ung1*::*URA3* cassette
OT-ung1-A	TAAGTTGAATGCTCCAGTGTTC	
OT-ung1-S	GGAGTCGTGACCATTCTACCT	To verify integration of the *ung1*::*URA3* cassette
In-ung1-S	CACCAGCACCACCGTCATTA	
In-ung1-A	CCAGGAACAACACTCCAGTCTA	
o1-rad30-S	GTATAAACCACTTATTGTAGTCTTCTAGCGC AGGCCTGCTCGCTGCAGGTCGACAACCC	For construction of the *rad30*::*URA3* cassette by amplifying the *URA3* fragment from plasmid pUG72 with long primers carrying homologous arms
o2-rad30-A	AGGACGTTTTAGTTGCTGAAGCCATATAATT GTCTATTTGGAGTGGATCTGATATCACC	
OT-rad30-S	GCTACCTAATCCTGCCGATCA	To verify integration of the *rad30*::*URA3* cassette
In-rad30-S	GGATACGATTCAGGAAGCACTA	
In-rad30-A	CGCCTAAGGTCCAGAATGATG	
o1-APN1-S	ACGCAACATTAATAAGCTTTTGGCATATCGG AACCATCGTAGCTGCAGGTCGACAACCC	For construction of the *apn1*::*URA3* cassette by amplifying the *URA3* fragment from plasmid pUG72 with long primers carrying homologous arms
o2-APN1-A	ACAAAAATTGATTACGTATTTAAAATTCTTCT CGCTTCTCAAGTGGATCTGATATCACC	
OT-apn1-S	TTGGTCCTTCACCTTGGCATA	To verify integration of the *apn1*::*URA3* cassette
IN-APN1-S	ATCTGAACAGGTGTGAGCAAT	
IN-APN1-A	TGATTCCAACCACTCCATAAGT	

o1-rad51-S	CGTAGTTATTTGTTAAAGGCCTACTAATTTGT TATCGTCATGCTGCAGGTCGACAACCC	For construction of the *rad51*::*URA3* cassette by amplifying the *URA3* fragment from plasmid pUG72 with long primers carrying homologous arms
o2-rad51-A	AGTAAACCTGTGTAAATAAATAGAGACAAG AGACCAAATACAGTGGATCTGATATCACC	
OT-rad51-S	TTATCACCGTCTCACCATATCC	To verify integration of the *rad51*::*URA3* cassette
in-rad51-S	GGTGGCGGTGAAGGTAAGT	
in-rad51-A	GGTAAGCAAGGTGAGTCAACAA	
OT-rad52-S	CCTTGGCGTCTGTATCATCTG	For construction of the *rad52*::*URA3* cassette
OT-rad52-A	CCGATGGAACTCAATGAACCTA	
OT2-rad52-S	ATAACGGTGAGTGTGGCAAC	To verify integration of the *rad52*::*URA3* cassette
in-rad52-S	CCGACTCGACGAAGAACCT	
in-rad52-A	CCTAATGGATTGTGCCTGGTAT	
OT-ctf18-S	TTTAGGGCGATGAAGGTTTGG	For construction of the *ctf18*::*URA3* cassette
OT-ctf18-A	AGTGGAGTTAATTCAGCAGTCT	
OT2-ctf18-S	CTTAGTTCTGCCAATGCCTTCT	To verify integration of the *ctf18*::*URA3* cassette
in-ctf18-S	CCTACTAGAGCGATTGAACCTT	
in-ctf18-A	GCGGAGTAACGTAACAATTCG	
OT-chl1-S	TATCCTTCCGCAGCATGAATG	For construction of the *chl1*::*URA3* cassette
OT-chl1-A	ACATCCACCACGAATAACACG	
OT2-chl1-S	TTGCCTGCCTGACTTCTTAGA	To verify integration of the *chl1*::*URA3* cassette
in-chl1-S	TGTGCGGACCTTAGACATAGTA	
in-chl1-A	CGGGATTGAGCCTGGACTT	
T-APN1-S	TCTAAGGAATTCACCATGGATCCTAGGGCCA TGCCTTCGACACCTAGC	For amplification of the *APN1* CDS, with 5′ extensions homologous to the pSH86 vector backbone
T-APN1-A	TACCCGGGTCGACGCGTAAGCTTGTGTTATT CTTTCTTAGTCTTCCTC	
pSH86-S	CACAAGCTTACGCGTCGAC	For amplification of the pSH86 vector backbone for construction of the *APN1* overexpression plasmid pSH86-APN1.
pSH86-A	GGCCCTAGGATCCATGGTGAAT	

Table S3. Summary of point mutations rates inEG0 (WT), DU, and derivative diploid strains.

Strain	Condition	Isolates	Total cell divisions	SNVs, n; rate ($\times 10^{-9}$)	InDels, n; rate ($\times 10^{-10}$)
EG0	0.005% gal	50	88500	697; 0.34 (0.32-0.37)	62; 0.31 (0.23-0.39)
DU	0.005% gal	29	8250	267; 1.41 (1.24-1.59)	53; 2.79 (2.09-3.65)
DUUΔung1	0.005% gal	15	3750	95; 1.10 (0.89-1.35)	5; 0.58 (0.19-1.35)
DUUΔapn1	0.02% gal	15	3750	687; 7.97 (7.38-8.58)	20; 2.32 (1.42-3.58)
DUUΔrev1	0.005% gal	13	3250	70; 0.94 (0.73-1.18)	57; 7.63 (5.78-9.88)
DUUΔrev3	0.005% gal	13	3250	87; 1.16 (0.93-1.43)	42; 5.62 (4.05-7.59)
DUUΔrad30	0.005% gal	15	3750	169; 1.96 (1.68-2.28)	38; 4.41 (3.12-6.05)
DU[a]	0.02% gal	10	2500	22; 0.38 (0.24-0.58)	3; 0.52 (0.11-1.52)
DU[b]	LG U− 0.005%gal	8	1000	55; 2.39 (1.89-3.11)	16; 6.96 (3.98-11.3)
DUU-APN1	LG U− 0.005%gal	9	1125	56; 2.16 (1.64-2.81)	11; 4.25 (2.12-7.61)

SNV and indel mutation rates were calculated as the number of observed SNVs or indels divided by the corresponding exposure (total cell divisions × diploid genome size). Values in parentheses indicate 95% confidence intervals (CI) calculated using a Poisson model.

Table S4. Summary of LOH, chromosomal rearrangements, and aneuploidy in EG0 (WT), DU, and derivative diploid strains

Strain	I-LOH, n; rate ($\times10^{-2}$)	T-LOH, n; rate ($\times10^{-2}$)	Del/Dup, n; rate ($\times10^{-3}$)	Aneuploidy, n; rate ($\times10^{-3}$)
EG0	251; 0.28 (0.25-0.32)	100; 0.11 (0.09-0.14)	14; 0.16 (0.09-0.27)	8; 0.09 (0.04-0.18)
DU	220; 2.67 (2.33-3.04)	235; 2.85 (2.50-3.24)	31; 3.76 (2.55-5.33)	26; 3.15 (2.06-4.62)
DUUΔung1	14; 0.37 (0.20-0.63)	5; 0.13 (0.04-0.31)	2; 0.53 (0.06-1.93)	18; 4.80 (2.84-7.59)
DUUΔapn1	248; 6.61 (5.82-7.49)	68; 1.81 (1.41-2.30)	4; 1.07 (0.29-2.73)	1; 0.27 (0.01-1.49)
DUUΔrev1	84; 2.58 (2.06-3.20)	105; 3.23 (2.64-3.91)	18; 5.54 (3.28-8.75)	14; 4.31 (2.36-7.23)
DUUΔrev3	84; 2.58 (2.06-3.20)	110; 3.38 (2.78-4.08)	15; 4.62 (2.58-7.61)	18; 5.54 (3.28-8.75)
DUUΔrad30	56; 1.49 (1.13-1.94)	103; 2.75 (2.24-3.33)	11; 2.93 (1.46-5.25)	12; 3.20 (1.65-5.59)
DU[a]	16; 0.64 (0.37-1.04)	7; 0.28 (0.11-0.58)	1; 0.40 (0.01-2.23)	0; 0.00 (0.00-1.48)
DU[b]	44; 4.40 (3.20-5.91)	58; 5.80 (4.40-7.50)	5; 5.00 (1.62-11.7)	12; 12.0 (6.20-21.0)
DUU-APN1	43; 3.82 (2.77-5.15)	82; 7.19 (5.80-9.47)	8; 7.11 (3.08-14.0)	21; 18.7 (11.6-28.5)

I-LOH, interstitial loss of heterozygosity; T-LOH, terminal loss of heterozygosity; Del/Dup, deletions/duplications. LOH and chromosomal alteration rates were calculated as the number of observed events divided by total cell divisions (Table S3). Values in parentheses indicate 95% Poisson-based confidence intervals.

Table S5. Associations of genomic elements with breakpoints of LOH events in DU MA isolates.

Analysis of associations of chromosomal elements with LOH events in DU strains.								
Chromosome elements	Predicted no. of elements involved in whole-genome sequencing	Observed no. of elements within LOH windows	Observed no. of elements outside LOH windows	Expected no. of elements within LOH windows	Expected no. of elements outside LOH windows	P value	Bonferroni-adjusted P value	Ratio of observed : expected
ARS elements (SGD)	352	221	9987	238	9970	0.265	5.032	0.93
Centromeres	16	18	446	10	454	0.011	0.200	1.80
G4 sequences (motif1)	538	342	15260	364	15238	0.243	4.622	0.94
Long terminal repeats (LTR)	288	196	8156	195	8157	0.942	17.902	1.01
Tandem repeat regions	1122	752	31786	760	31778	0.769	14.612	0.99
Replication-termination (ter) sequences	71	64	1995	48	2011	0.019	0.370	1.33
Transposable elements (Ty)	50	40	1410	33	1417	0.218	4.136	1.21
Regions with high levels of gamma-H2AX	629	442	17799	426	17815	0.433	8.223	1.04
Regions of high G C content (2kb,0.42)	570	539	15991	386	16144	0.000	0.000	1.40
Highly-transcribed genes	330	229	9341	223	9347	0.684	13.002	1.03
Regions of low G C content (2kb,0.36)	890	617	25193	603	25207	0.564	10.716	1.02
Meiotic hotspots	136	70	3874	92	3852	0.020	0.386	0.76
ncRNA genes	30	32	838	20	850	0.007	0.126	1.60
Palindromic sequences	570	343	16187	386	16144	0.027	0.509	0.89
Binding sites for Rrm3p	112	107	3141	75	3173	0.000	0.004	1.43
snoRNA genes	79	96	2195	53	2238	0.000	0.000	1.81
tRNA genes	335	223	9492	227	9488	0.788	14.976	0.98
PoorNER	142	114	4004	96	4022	0.063	1.198	1.19
Weakly-transcribed genes	312	220	8828	211	8837	0.531	10.083	1.04

P values were calculated using a chi-square test and adjusted for 19 comparisons using the Bonferroni method.

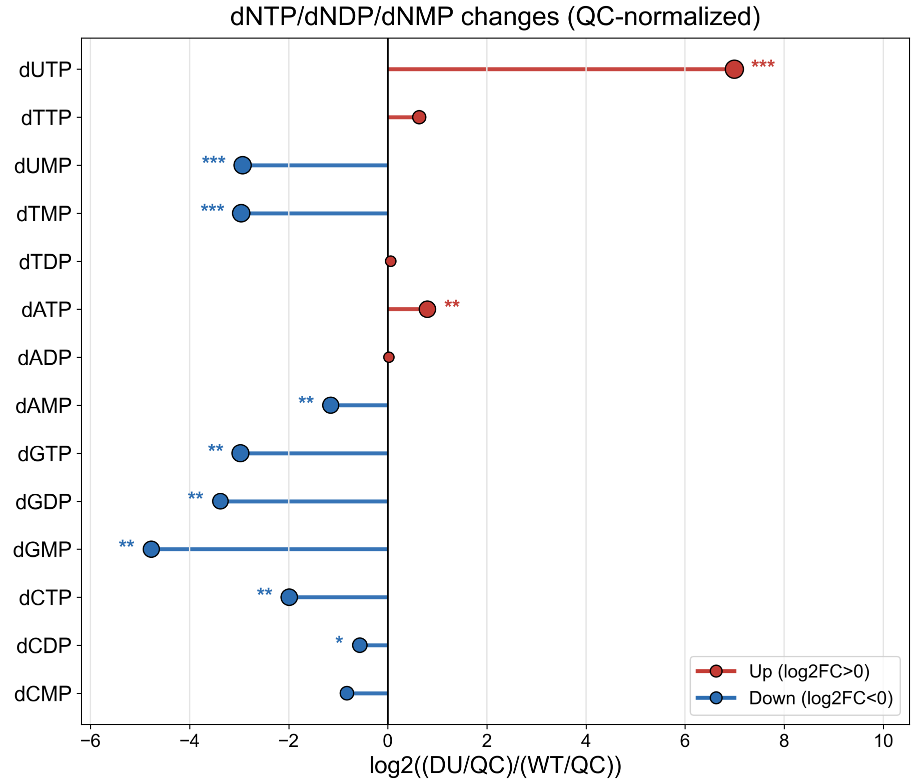

Fig. S1. Changes in deoxyribonucleotide pools after DUT1 downregulation. Intracellular deoxyribonucleotide levels were measured by targeted metabolomics. Values were normalized to the mean of quality control (QC) samples within each batch and are shown as log2 fold changes in DU relative to WT (EG0). Red and blue indicate increased and decreased abundance in DU, respectively. Dot size represents −log10(*P* value). Statistical significance was assessed using Welch's t test. **P* < 0.05, ***P* < 0.01, ****P* < 0.001.

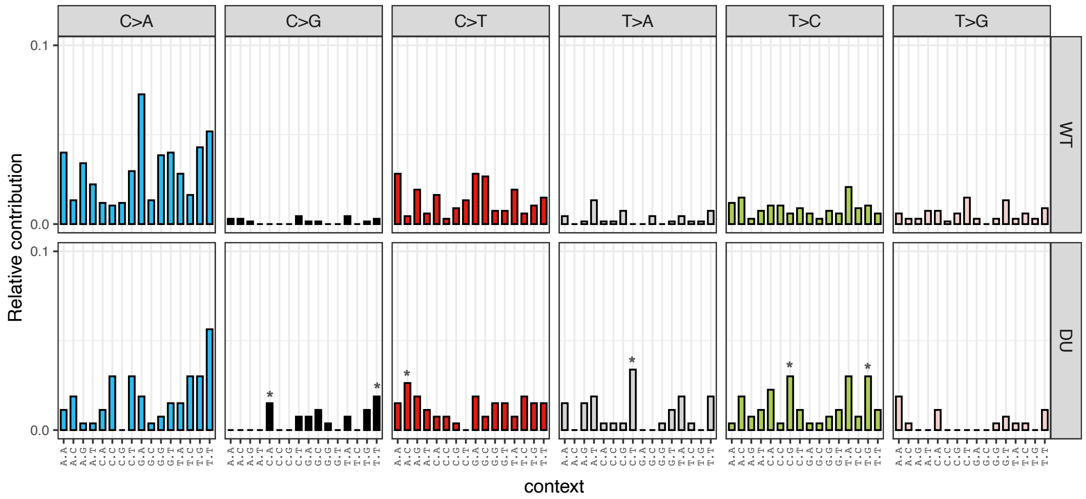

Fig. S2. Mutational context profiles of SNVs in WT (EG0) and DU isolates. Relative contributions of the 96 trinucleotide mutation contexts in WT and DU mutation-accumulation isolates. SNVs are grouped by the six base-substitution classes, with the mutated base shown in the central position of each trinucleotide context. The y-axis indicates the relative contribution of each context within the total SNV set. Asterisks indicate contexts with significantly different proportions between WT and DU, as assessed by Fisher's exact test. **P* < 0.05.

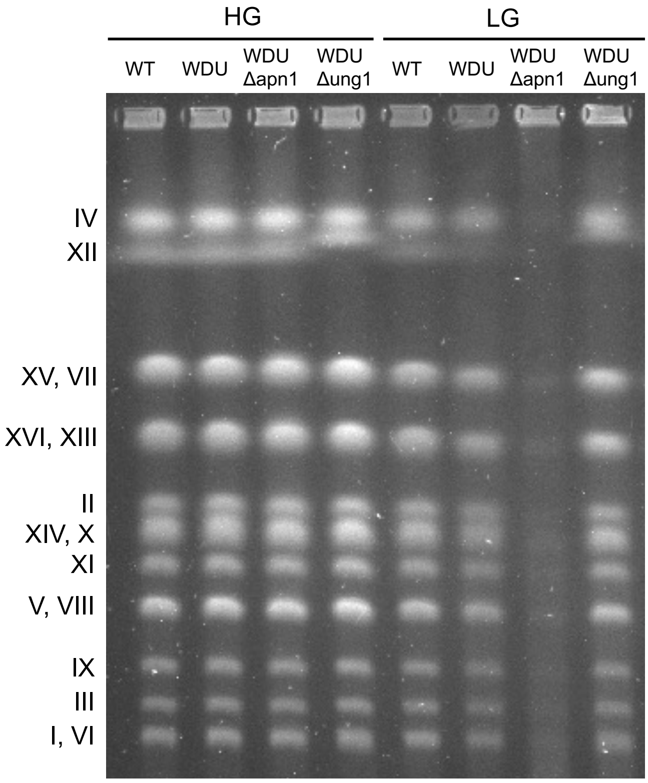

Fig. S3. PFGE reveals chromosome fragmentation under *DUT1*-limiting conditions. WT (SY165), WDU, WDUΔapn1 and WDUΔung1 cells were cultured in HG or LG YPR medium. All strains shared the W303-1A genetic background. Chromosome-sized DNA molecules were separated by pulsed-field gel electrophoresis.

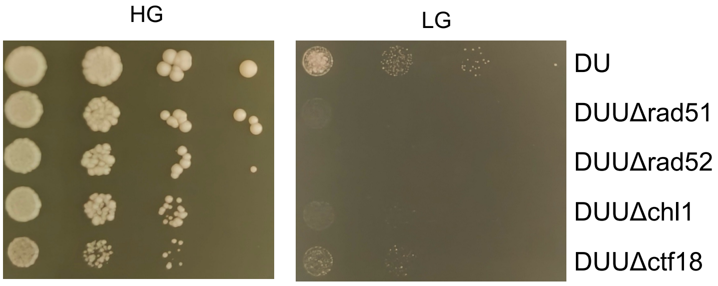

Fig. S4. Deletion of *RAD51*, *RAD52*, *CHL1*, or *CTF18* exacerbates growth defects under Dut1-limiting conditions. Tenfold serial dilutions of DU, DUUΔrad51, DUUΔrad52, DUUΔchl1, and DUUΔctf18 cells were spotted onto HG or LG YPR plates and incubated at 30°C. All strains grew under HG conditions. Under LG conditions, deletion of *RAD51*, *RAD52, CHL1*, or *CTF18* markedly impaired growth relative to DU.

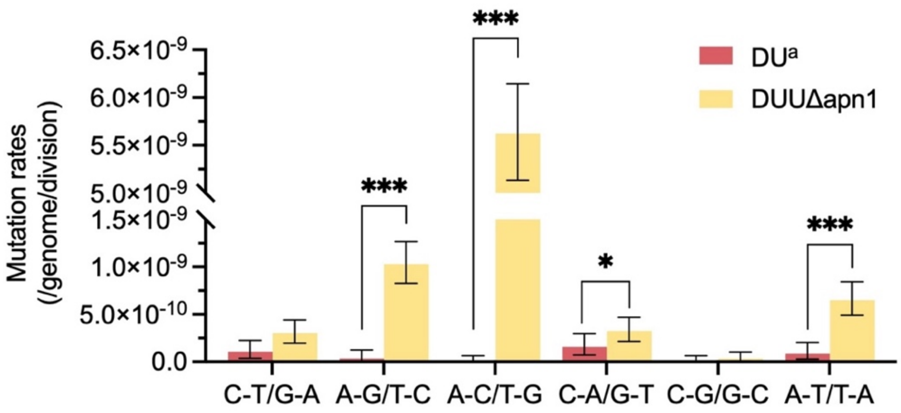

Fig. S5. ***APN1*** **deletion alters the SNV spectrum under reduced dUTPase expression.** Rates of individual SNVs classes in DU^a and DUUΔapn1 mutation-accumulation isolates, shown as complementary base-substitution pairs. DU^a denotes DU isolates propagated on 0.02% galactose plates. Error bars indicate 95% Poisson confidence intervals. Statistical significance is indicated as *$P < 0.05$, **$P < 0.01$, ***$P < 0.001$. P values were calculated using the Wilcoxon rank-sum test.

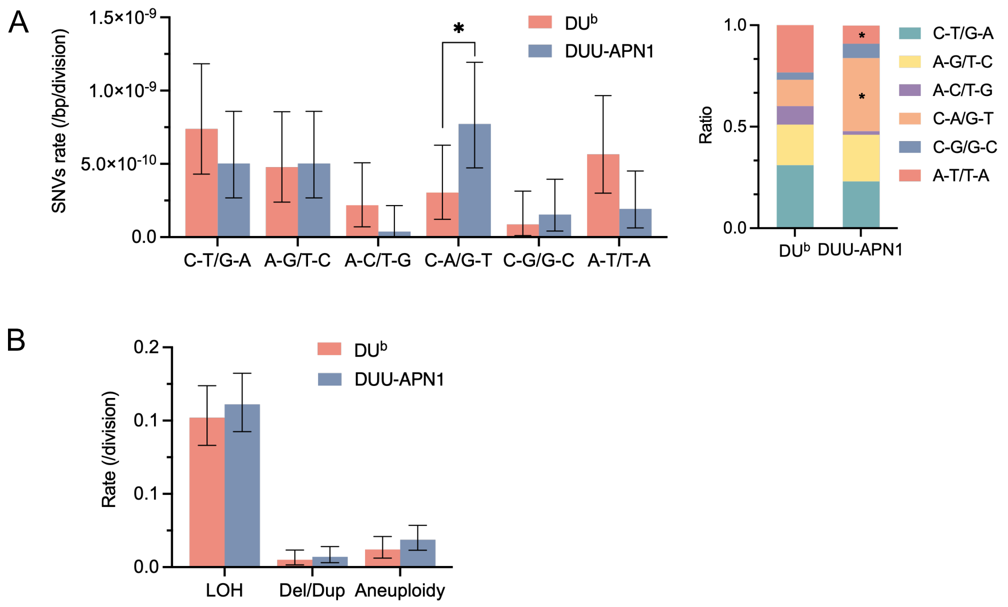

Fig. S6. *APN1* overexpression has limited effects on genome instability under reduced *DUT1* expression. (A) Rates of individual SNVs classes in DU^b and DUU-APN1 isolates, shown as complementary base-substitution pairs. Relative ratios of the six base-substitution classes in DU^b and DUU-APN1 isolates are shown in the right part. (B) Rates of LOH, large deletions/duplications (Del/Dup) and aneuploidy in DU^b and DUU-APN1 isolates. Error bars in panels A and B indicate 95% Poisson confidence intervals. Statistical significance is indicated as $*P < 0.05$. P values were calculated using the Wilcoxon rank-sum test.

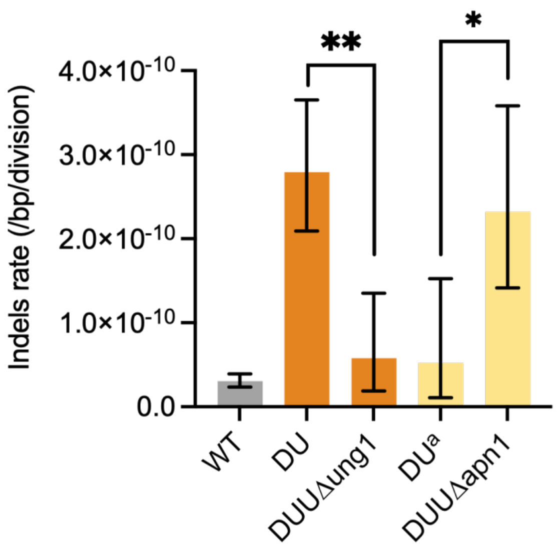

Fig. S7. Effects of BER gene deletions on small indel formation under reduced dUTPase expression. Genome-wide indel rates in WT, DU, DUUΔung1, DU[a] and DUUΔapn1 mutation-accumulation isolates. DU[a] denotes DU isolates propagated on 0.02% galactose plates. Error bars indicate 95% Poisson confidence intervals. Statistical significance is indicated as *$P < 0.05$, **$P < 0.01$. P values were calculated using the Wilcoxon rank-sum test.
